# 2-Cys peroxiredoxins contribute to thylakoid lipid unsaturation by affecting ω-3 fatty acid desaturase 8

**DOI:** 10.1093/plphys/kiae102

**Published:** 2024-02-22

**Authors:** María Luisa Hernández, Julia Jiménez-López, Francisco Javier Cejudo, Juan Manuel Pérez-Ruiz

**Affiliations:** Departamento de Bioquímica Vegetal y Biología Molecular, Instituto de Bioquímica Vegetal y Fotosíntesis , Universidad de Sevilla and CSIC, Avda. Américo Vespucio, 49, 41092 Sevilla, Spain; Departamento de Bioquímica Vegetal y Biología Molecular, Instituto de Bioquímica Vegetal y Fotosíntesis , Universidad de Sevilla and CSIC, Avda. Américo Vespucio, 49, 41092 Sevilla, Spain; Departamento de Bioquímica Vegetal y Biología Molecular, Instituto de Bioquímica Vegetal y Fotosíntesis , Universidad de Sevilla and CSIC, Avda. Américo Vespucio, 49, 41092 Sevilla, Spain; Departamento de Bioquímica Vegetal y Biología Molecular, Instituto de Bioquímica Vegetal y Fotosíntesis , Universidad de Sevilla and CSIC, Avda. Américo Vespucio, 49, 41092 Sevilla, Spain

## Abstract

Fatty acid unsaturation levels affect chloroplast function and plant acclimation to environmental cues. However, the regulatory mechanism(s) controlling fatty acid unsaturation in thylakoid lipids is poorly understood. Here, we have investigated the connection between chloroplast redox homeostasis and lipid metabolism by focusing on 2-Cys peroxiredoxins (Prxs), which play a central role in balancing the redox state within the organelle. The chloroplast redox network relies on NADPH-dependent thioredoxin reductase C (NTRC), which controls the redox balance of 2-Cys Prxs to maintain the reductive activity of redox-regulated enzymes. Our results show that Arabidopsis (*Arabidopsis thaliana*) mutants deficient in 2-Cys Prxs contain decreased levels of trienoic fatty acids, mainly in chloroplast lipids, indicating that these enzymes contribute to thylakoid membrane lipids unsaturation. This function of 2-Cys Prxs is independent of NTRC, the main reductant of these enzymes, hence 2-Cys Prxs operates beyond the classic chloroplast regulatory redox system. Moreover, the effect of 2-Cys Prxs on lipid metabolism is primarily exerted through the prokaryotic pathway of glycerolipid biosynthesis and fatty acid desaturase 8 (FAD8). While 2-Cys Prxs and FAD8 interact in leaf membranes as components of a large protein complex, the levels of FAD8 were markedly decreased when *FAD8* is overexpressed in 2-Cys Prxs-deficient mutant backgrounds. These findings reveal a function for 2-Cys Prxs, possibly acting as a scaffold protein, affecting the unsaturation degree of chloroplast membranes.

## Teaser

2-Cys peroxiredoxins participate in lipid metabolism independently of their function in chloroplast redox homeostasis.

## Introduction

Chloroplasts, the specific organelles of plants and algae that perform oxygenic photosynthesis, contain an extensive internal membrane network, the thylakoid membranes, in which pigments, proteins, and redox-active cofactors are assembled to perform the photochemical and electron transport reactions of photosynthesis. The lipid composition of chloroplast membranes is unique and highly conserved, having glycolipids as the main constituents, in contrast with extraplastidial membranes, which are enriched in phosphoglycerolipids (Hölzl and Dörmann 2019). The galactolipids monogalactosyldiacylglycerol (MGDG) and digalactosyldiacylglycerol (DGDG) are the most abundant lipids in chloroplast membranes, accounting for about 50% and 25% of total thylakoid lipids, respectively. In lower proportion, chloroplast membranes contain the anionic lipids sulfoquinovosyldiacylglycerol (SQDG) and phosphatidylglycerol (PG), the most abundant phospholipid in thylakoids and inner envelope membranes (Hölzl and Dörmann 2019). An additional specific feature of chloroplast membrane lipids is their distinctive fatty acid composition. Trienoic fatty acids (TA), α-linolenic (18:3) or hexadecatrienoic (16:3) acids, typically account for approximately 70% of all fatty acids and over 90% of the MGDG, the major thylakoid lipid. Moreover, a unique fatty acid is located in *sn*-2 position of PG, the 3E-hexadecenoic acid (16:1t), which contains a *trans* double bond in Δ3 position of the carbon chain, while most double bonds in plant fatty acids are in the *cis* configuration (Gao et al. 2009).

In vascular plants, de novo fatty acid biosynthesis occurs in plastids where the fatty acid synthase complex produces 16 (C-16) or 18 (C-18) carbon fatty acids. Subsequently, C-18 fatty acids can be desaturated by the soluble stearoyl-ACP desaturase to oleoyl-ACP, which is the major product of plastid fatty acid synthesis. These products are either used by the prokaryotic pathway for the synthesis of glycerolipids within the chloroplast or exported to the cytosol where they are incorporated to the endoplasmic reticulum (ER) for glycerolipid assembly. Phospholipids assembled in the ER can return to the chloroplast envelope to be used as precursors for galactolipids biosynthesis via the eukaryotic pathway (Li-Beisson et al. 2013). In 18:3 plants, such as legumes and monocots, only 18:3 acyl groups are present at *sn*-2 position of galactolipids as they are exclusively derived from the eukaryotic pathway. In contrast, in 16:3 plants, like Arabidopsis (*Arabidopsis thaliana*), the prokaryotic and eukaryotic pathways contribute to galactolipid biosynthesis, while chloroplast PG, in all plants, is mostly synthesized from phosphatidic acid (PA) via the prokaryotic pathway (Hurlock et al. 2018). In both biosynthetic pathways, further desaturation occurs in fatty acids esterified to membrane lipids by membrane bound fatty acid desaturases (FAD), which differ in cellular localization, substrate specificity, and electron donor system (Shanklin and Cahoon 1998). The microsomal ω-6 (FAD2) and ω-3 (FAD3) desaturases are located in the ER while ω-6 (FAD6) and ω-3 (FAD7/8) are present in the chloroplast.

The unique chloroplast membrane system plays an important function in chloroplast biogenesis and performance (Kobayashi 2016; Hernández and Cejudo 2021), being the redox state of the organelle a likely mechanism involved in the coordination of photosynthesis and lipid metabolism (Geigenberger and Fernie 2014; Yu et al. 2020). Chloroplasts harbor a complex redox network in which the NADPH-dependent thioredoxin reductase C (NTRC) controls the redox balance of 2-Cys peroxiredoxins (Prxs), thiol-dependent peroxidases that maintain the reductive activity of chloroplast thioredoxins (Trxs) (Pérez-Ruiz et al. 2017). Thereby, the NTRC–2-Cys Prx system adjusts the redox state of chloroplast enzymes in response to light and darkness (Cejudo et al. 2019). The 2-Cys Prxs present in Arabidopsis chloroplast, A (At3g116309) and B (At5g06290), are dimeric thiol-dependent peroxidases in which the catalytic cysteine residues, peroxidatic (C_P_) and resolving (C_R_), that participate in the catalytic cycle, are located in different monomers (Dietz 2011; Liebthal et al. 2018), and are among the most abundant proteins of the organelle (Peltier et al. 2006). Additional thiol-dependent peroxidases in the chloroplast Prxs Q (At3g26060) and IIE (At3g52960) are monomeric, thus containing the C_P_ and C_R_ cysteines in the same polypeptide (Dietz 2011; Liebthal et al. 2018). These Prxs efficiently reduce H_2_O_2_ and other organic peroxides, participating in antioxidant defense in photosynthesis and stress response (Dietz 2016). Fatty acid desaturation has been linked to the redox regulatory network as an adaptative response to environmental cues (Yu et al. 2020). Additionally, the soluble stearoyl-ACP desaturase has been identified as a redox-sensitive protein (Liu et al. 2014), and FAD4, responsible for the unusual 16:1t synthesis in PG, was recently reported to require Prx Q for its activity (Horn et al. 2020), thereby linking PG-16:1t to a signaling process that perceives the redox state of the chloroplast in response to stress (Hoh et al. 2022). These findings suggest the relevant role of redox regulation on lipid metabolism (Geigenberger and Fernie 2014; Hernández and Cejudo 2021), however, the molecular basis of this mechanism remains poorly understood. Here, we have investigated the influence of the chloroplast redox state on lipid metabolism by analyzing the contribution of 2-Cys Prxs, which balance the redox state of the chloroplast, to the membrane lipid composition of the organelle. Our results uncover a function of 2-Cys Prxs in lipid metabolism as the content of these enzymes determines the level of thylakoid lipids unsaturation, primarily in the prokaryotic pathway of glycerolipids biosynthesis. This function of 2-Cys Prx is exerted independently of NTRC, the main reductant of these enzymes. Moreover, we observed that the levels of FAD8 in Arabidopsis transgenic plants depends on the contents of 2-Cys Prxs, both enzymes interacting in a supramolecular complex in leaf membranes. These results suggest that 2-Cys Prxs contribute to the level of fatty acid unsaturation in chloroplasts by affecting FAD8 stability.

## Results

### 2-Cys Prxs modulate the biosynthesis of TA by chloroplast ω-3 desaturases in Arabidopsis leaves

**Figure 1.**
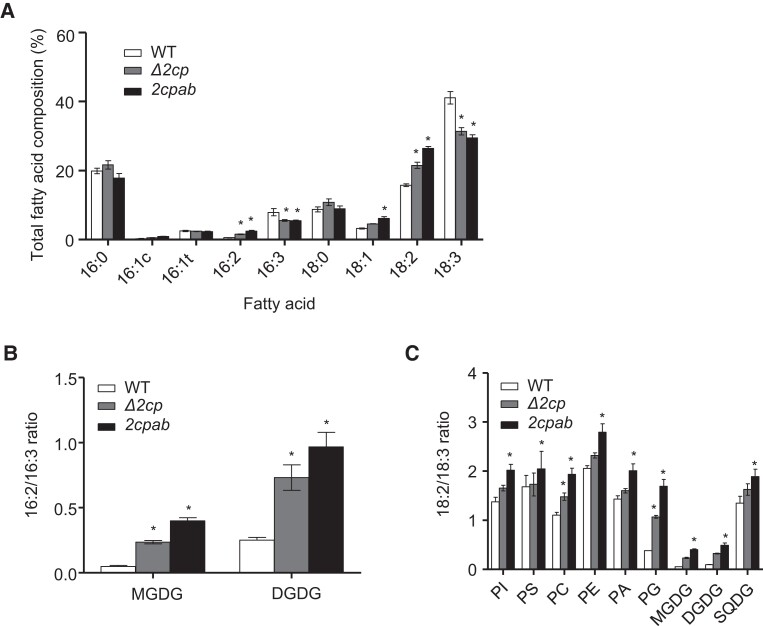
Lipid profiling of Arabidopsis leaves from WT and 2-Cys Prxs deficient lines. WT and the mutants *Δ2cp* and *2cpab* were grown under LD photoperiod for 4 wk. Fatty acid composition of total lipids (**A**) and dienoic to trienoic fatty acids ratios, 16:2/16:3 in galactolipids (**B**), and 18:2/18:3 ratio in polar lipids classes (**C**) were analyzed in rosette leaves from plants grown as stated above. Total leaf lipids were extracted, the different lipid classes separated by TLC and fatty acid compositions analyzed as described in Materials and Methods. Indicated lipids are DGDG, MGDG, PA, PC, PE, PG, PI, phosphatidylserine (PS), and SQDG. Data are means ± Sd of three independent plants. Asterisks indicate significantly different (*P* < 0.05) to WT according to two-way ANOVA with a Bonferroni posttest.

To investigate the influence of chloroplast redox systems on lipid metabolism, we analyzed the leaf fatty acid composition in Arabidopsis mutant lines either partially or completely devoid of 2-Cys Prxs, the double mutant *delta 2-cys prx* (*Δ2cp*) (Pulido et al. 2010), containing ∼5% of wild-type (WT) levels of 2-Cys Prxs (no 2-Cys Prx B and residual amounts of 2-Cys Prx A) and the double null mutant *2cpab* (Ojeda et al. 2018), respectively. Remarkably, the levels of 16:3 and 18:3 TA were significantly lower in *Δ2cp* (∼5.5% and ∼31.4%) and *2cpab* (∼5.4% and ∼29.4%) mutant leaves than in the WT (∼7.9% and ∼41.1%) (Fig. 1A), while both mutants showed increased levels of dienoic fatty acids (DA), 16:2 and 18:2. Remaining fatty acid species exhibited similar levels in the mutants and the WT, with the exception of oleic acid (18:1), which was slightly increased in *2cpab* mutant leaves (Fig. 1A). In contrast, single T-DNA insertion mutants of Arabidopsis deficient in either 2-Cys Prxs A (Pérez-Ruiz et al. 2017) or B (Kirchsteiger et al. 2009) did not show remarkable differences in the levels of 16:3 and 18:3 TA compared to WT leaves (Supplementary Fig. S1), as previously reported (Horn et al. 2020). These results suggest that 2-Cys-Prxs exert a positive dose-dependent effect on the conversion of DA into TA, a reaction catalyzed by ω-3 fatty acid desaturases, i.e. FAD3, FAD7, and FAD8. To confirm this possibility, a complementation test was performed by generating transgenic lines (*2cpab*/2CPA-OE) expressing the coding sequence of the *2-Cys PRX A* gene under the control of the cauliflower mosaic virus (CaMV) 35S promoter in the *2cpab* mutant background. As controls, Arabidopsis transgenic lines expressing a mutant variant of 2-Cys Prx A in which the peroxidatic cysteine (C_P_) residue at the active site of the enzyme was replaced by Ser (*2cpab*/2CPA-C_P_-S-OE) were also generated. Immunoblot analysis showed that the WT and the mutant variant of 2-Cys Prx A, 2CPA, and 2CPA-C_P_-S, respectively, accumulated at similar levels in *2cpab*/2CPA-OE and *2cpab*/2CPA-C_P_-S-OE transgenic plants. Expectedly, the levels of dimeric enzyme were very reduced in the 2CPA-C_P_-S mutant variant as the formation of intermolecular disulfide bonds is impaired (Supplementary Fig. S2A). DA-to-TA ratios, 16:2/16:3 and 18:2/18:3, in *2cpab*/2CPA-OE plants showed similar values to those of the WT, indicating complementation of the TA deficiency phenotype of the *2cpab* mutant (Supplementary Fig. S2, B and C). On the contrary, *2cpab*/2CPA-C_P_-S-OE plants displayed ratios comparable to those of the *2cpab* mutant (Supplementary Fig. S2, B and C), indicating that the effect of 2-Cys Prx A on the conversion of DA into TA depends on the functional form of the enzyme.

To further investigate the effect of 2-Cys Prxs on lipid biosynthesis, we determined lipid profiles of rosette leaves from the *Δ2cp* and *2cpab* mutants. Overall, the composition of polar lipids in leaves from the mutants was like that of the WT, except for MGDG, which was slightly reduced in both *Δ2cp* and *2cpab* mutants (Supplementary Fig. S3). On the contrary, the fatty acid composition of leaf lipids was remarkably altered in 2-Cys Prxs-deficient plants. DA/TA ratios were markedly increased in all lipid classes in the *2cpab* knockout mutant whereas in the less severe *Δ2cp* knockdown mutant significant differences were observed only for 16:2/16:3 in MGDG and DGDG and 18:2/18:3 in phosphatidylcholine (PC) and PG (Fig. 1, B and C). Given that the levels of TA, primarily in thylakoid lipids (Fig. 1, A to C), correspond with the severity of the mutant alleles for 2-Cys Prxs, we next analyzed DA/TA ratios in main thylakoid membrane lipids in transgenic lines (2CPA-OE) expressing 2-Cys Prx A under the control of the 35S promoter in the WT background (Pérez-Ruiz et al. 2017). Western blot analysis showed the expected over-accumulation of 2-Cys Prxs in 2CPA-OE plants compared to the WT (Supplementary Fig. S4A). Nevertheless, the DA/TA ratios in PG, MGDG, and DGDG were like those of the WT in the 2CPA-OE plants (Supplementary Fig. S4, B and C), indicating that increased amounts of 2-Cys Prxs do not affect TA levels in leaves. In addition, the effect of the disruption of 2-Cys Prxs on ω-3 desaturation in the main thylakoid lipids was even more severe under short-day (SD) photoperiod. Particularly, 16:2/16:3 ratios in MGDG and DGDG and 18:2/18:3 ratio in PG significantly increased when *2cpab* mutant plants were grown under SD conditions (Supplementary Table S1), indicating that the altered unsaturation lipid phenotype of the *2cpab* mutant is photoperiod-dependent, in line with other phenotypic features reported for this mutant, which are aggravated under these growth conditions (Ojeda et al. 2018). Taken together, these results indicate that 2-Cys Prxs mainly affect the function of plastidial ω-3 desaturases, FAD7 and/or FAD8, although a contribution of 2-Cys Prxs to the microsomal FAD3 cannot be dismissed.

### The role of 2-Cys Prxs in lipid metabolism is independent of ROS detoxification and NTRC-mediated redox regulation

**Figure 2.**
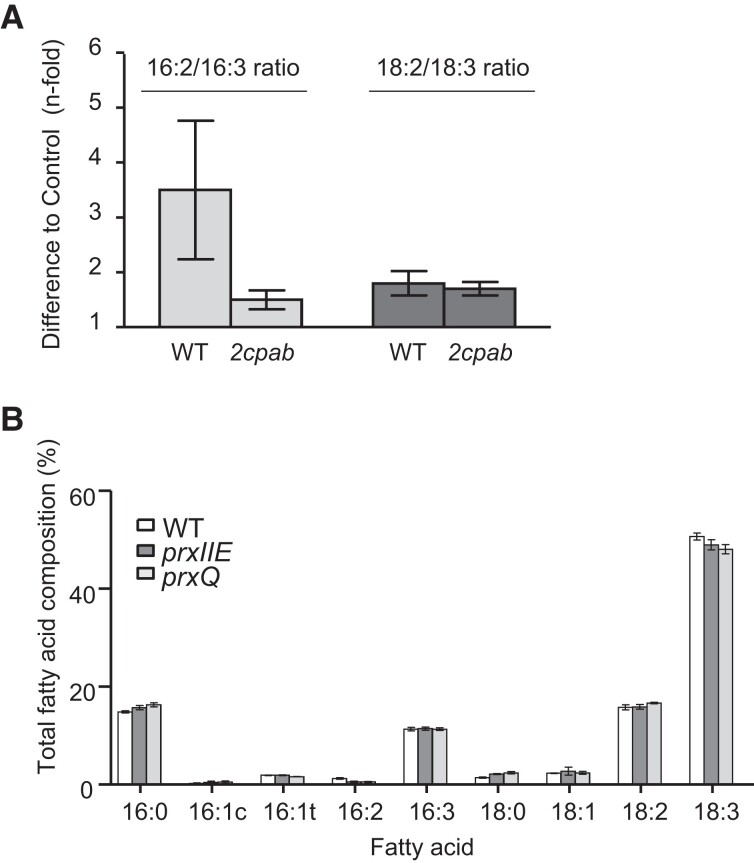
Effect of disturbed ROS metabolism on trienoic acids levels of Arabidopsis leaves. **A)** Dienoic to trienoic acids ratios, 16:2/16:3 and 18:2/18:3, in rosette leaves from WT and *2cpab* plants grown under SD photoperiod (light intensity of 125 *µ*E m^−2^ s^−1^) for 6 wk (control) and exposed for an additional week to high (930 *µ*E m^−2^ s^−1^) and continuous light. Values represent the *n*-fold differences in the 16:2/16:3 and 18:2/18:3 ratios of the HL treatment compared to control conditions. **B)** Fatty acid composition of total lipids of rosette leaves from WT and the *prxIIE* and *prxQ* mutant plants grown under LD conditions (light intensity of 125 *µ*E m^−2^ s^−1^) for 4 wk. Total leaf lipids were extracted, and fatty acid composition analyzed as described in Material and Methods. Data are means ± Sd of three independent plants.

Given the hydrogen peroxide scavenging activity of 2-Cys Prxs (Liebthal et al. 2018), the altered lipid metabolism of the *Δ2cp* and *2cpab* mutants could be explained by the impaired antioxidant activity in these plants. In line with this notion, it was previously reported that the *2cpab* mutant is sensitive to high-light conditions due to lower PSII photochemical efficiency and the accumulation of superoxide anion radicals and H_2_O_2_ (Awad et al. 2015). Thus, to test whether the effect of 2-Cys Prxs on the levels of TA in leaves is due to the antioxidant activity of these enzymes, we compared the fatty acid composition in WT and *2cpab* plants exposed to high light (HL, 930 *µ*E m^−2^ s^−1^) (Supplementary Table S2). This treatment was conducted using SD grown plants to prevent HL-induced flowering. Notably, the increase in the 18:2/18:3 ratio observed in WT leaves exposed to HL in comparison to control conditions, was like that of the *2cpab* mutant (Fig. 2A). Instead, the effect of HL on the 16:2/16:3 was even less severe in the *2cpab* mutant, although it is important to note that the decrease of 16:3 content by the HL conditions was compensated by an increase in palmitic acid (16:0) in both lines (Supplementary Table S2), suggesting that additional FADs are affected by this treatment. Arabidopsis chloroplast harbor additional Prxs, such as Prx Q and Prx IIE, which might influence fatty acid unsaturation. To test this possibility, we analyzed the fatty acid composition in mutant plants deficient in Prx Q, the *prxq* mutant (Lamkemeyer et al. 2006), or Prx IIE, the *prxIIE* mutant (Romero-Puertas et al. 2007). Both showed almost identical fatty acid composition than the WT (Fig. 2B). Overall, these results indicate that the most relevant effect on TA synthesis is exerted by 2-Cys Prxs, this effect being probably unrelated to the impaired antioxidant capacity of these plants.

**Figure 3.**
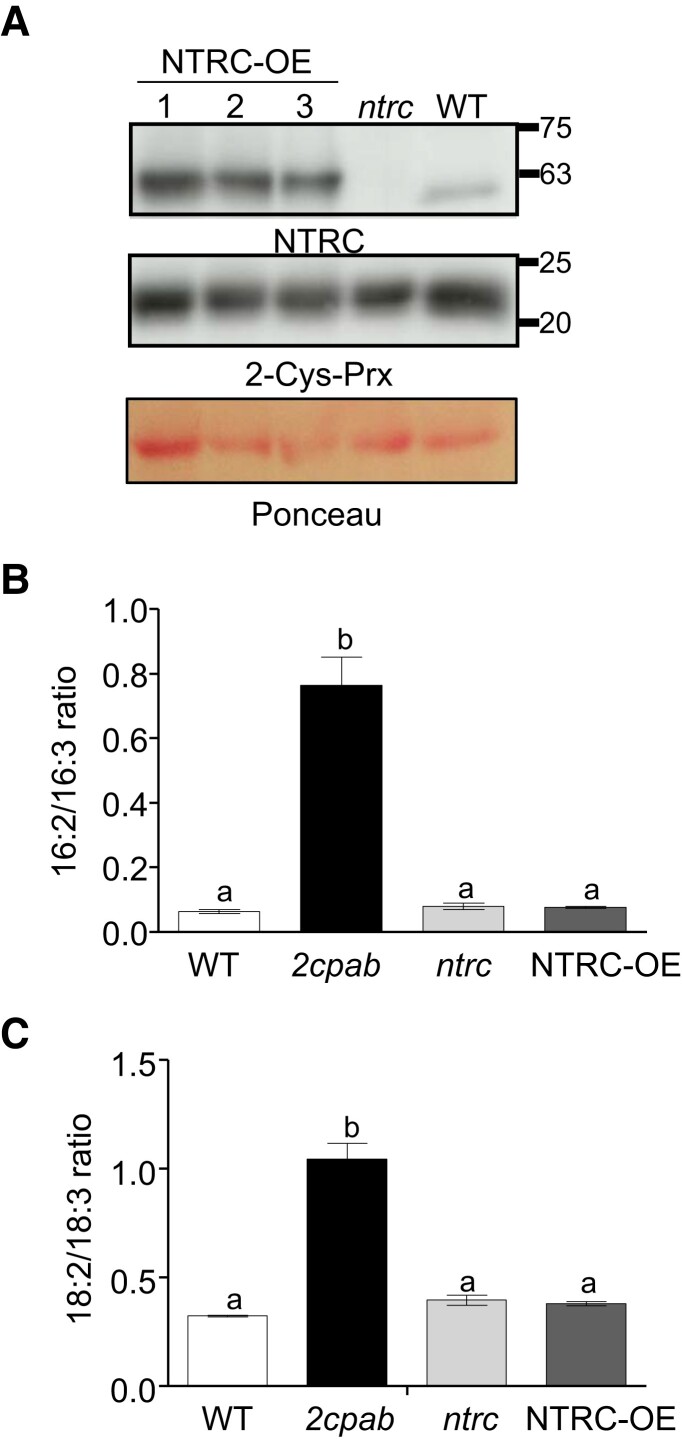
Effect of NTRC-mediated chloroplast redox imbalance on trienoic acid levels of Arabidopsis leaves. WT, the *ntrc* mutant, and a transgenic line overexpressing (OE) NTRC in the WT background (NTRC-OE) were grown under SD photoperiod for 7 wk. **A)** Western blot analysis of the levels of NTRC and 2-Cys Prxs in WT, *ntrc* and three (1, 2, and 3) individuals of NTRC-OE, as indicated. Protein extracts (15 *µ*g) were subjected to SDS–PAGE under reducing conditions, transferred to nitrocellulose filters, and probed with anti-NTRC and anti-2-Cys Prxs antibodies. Even loading was monitored by Ponceau staining of the Rubisco large subunit. Molecular mass markers (kDa) are indicated on the right. Dienoic to trienoic fatty acids ratios, 16:2/16:3 **B**) and 18:2/18:3 (**C**), determined in rosette leaves from the indicated genotypes. Total leaf lipids were extracted, and fatty acid composition analyzed as described in Materials and Methods. Data are means ± Sd of four independent plants. Letters indicate significant differences by one-way ANOVA with a Tukey's multiple comparison test (*P* < 0.05).

The NTRC–2-Cys Prx redox couple plays a central role in maintaining chloroplast redox homeostasis, hence we then investigated whether altered TA synthesis in 2-Cys Prxs-deficient mutants was caused by impaired operation of this redox regulatory system. To that end, we analyzed the impact of NTRC, which maintains the redox balance of 2-Cys Prxs (Pérez-Ruiz et al. 2017), on lipid metabolism by analyzing fatty acid composition in plants either lacking NTRC, the *ntrc* mutant (Serrato et al. 2004), or overexpressing the enzyme, the NTRC-OE line (Ojeda et al. 2018). These determinations were carried out on plants grown under SD conditions (light intensity of 125 *µ*E m^−2^ s^−1^), in which the *2cpab* mutant displayed the most TA severe phenotype (Supplementary Table S2). Both, *ntrc* and NTRC-OE plants, which contained WT levels of 2-Cys Prxs (Fig. 3A), showed similar DA/TA ratios than the WT (Fig. 3, B and C), indicating that the redox mechanism by which 2-Cys Prxs affect lipid metabolism is independent of the NTRC-mediated chloroplast redox homeostasis.

### 2-Cys Prxs mainly affect the prokaryotic pathway of lipid synthesis through FAD8

**Figure 4.**
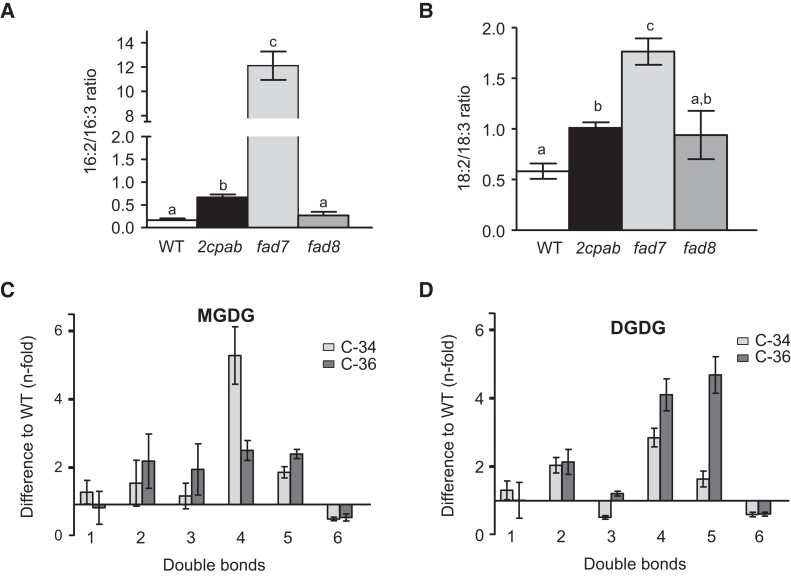
Comparative analysis of dienoic to trienoic fatty acid ratios in *2cpab, fad7* and *fad8* mutants and *2cpab* mutant lipidomic analysis. Dienoic to trienoic fatty acids ratios, 16:2/16:3 (**A)** and 18:2/18:3 (**B)**, measured in rosette leaves from WT, *2cpab*, *fad7* and *fad8* plants grown under LD photoperiod for 4 wk. Total leaf lipids were extracted, and fatty acid composition analyzed as described in Materials and Methods. Data are means ± Sd of three independent plants. Letters indicate significant differences by one-way ANOVA with a Tukey's multiple comparison test (*P* < 0.05). **C, D)** Lipidomic analysis of galactolipid molecular species in WT and *2cpab* rosette leaves. Galactolipid molecular species, MGDG and DGDG, were analyzed by ESI–MS/MS from plants grown under SD conditions for 7 wk, and different lipid classes were quantified in comparison to the signals for peaks of internal standard, as described in Materials and Methods. Data are means ± Sd of five independent plants. The *n*-fold differences in the amount of C34 and C36 MGDG (**C**) and DGDG (**D**) molecular species in *2cpab* mutant leaves compared to WT has been represented.

In Arabidopsis, chloroplast ω-3 desaturases are functionally nonredundant under standard growth conditions as FAD7 shows higher preference for the desaturation of 16:2 and 18:2 in MGDG and DGDG (Román et al. 2015), whereas FAD8 is highly specific for PG-18:2 (Browse et al. 1986; Román et al. 2015). Thus, the fact that the *2cpab* mutant displayed reduced 18:3 levels primarily in PG (Fig. 1C and Supplementary Table S1) points to FAD8 as the main ω-3 desaturase affected by the deficiency of 2-Cys Prxs. To test this possibility, we first compared DA/TA ratios of *2cpab* and single *fad7* and *fad8* mutant plants (Román et al. 2015). As expected, 16:2/16:3 and 18:2/18:3 ratios significantly increased in *fad7* (∼12.1 and ∼1.8) compared to *fad8* (∼0.3 and ∼0.9), which showed DA/TA ratios comparable to the WT (∼0.2 and ∼0.6), though 18:2/18:3 ratio was slightly increased (Fig. 4, A and B). Notably, the 16:2/16:3 ratio determined in the *2cpab* mutant (∼0.7) was much smaller than that of the *fad7* mutant, but significantly higher than those of the WT and *fad8* plants (Fig. 4A). Likewise, the 18:2/18:3 ratio determined in the *2cpab* mutant (∼1.0) was lower than that of the *fad7* mutant and very similar to that of the *fad8* mutant (Fig. 4B). Therefore, the absence of 2-Cys Prxs nearly mimicked the effect of the lack of FAD8 on the desaturation of 18:2 fatty acids, suggesting that these enzymes may be functionally related. Nevertheless, the fact that desaturation of 16:2 to 16:3 is still affected in the *2cpab* mutant, but not in the *fad8* mutant, indicates that the function of FAD7 could be also influenced, although at a lesser extent, by 2-Cys Prxs.

The above results suggest that 2-Cys Prxs exert a different impact on the prokaryotic and eukaryotic pathways of lipid synthesis, since FAD8 function is related to PG in the prokaryotic pathway, while FAD7 activity is related to MGDG and DGDG from both glycerolipid biosynthetic pathways (Browse et al. 1986; McConn et al. 1994). To address this issue, a lipidomic analysis of galactolipid molecular species was performed in rosette leaves of the *2cpab* mutant grown under SD photoperiod (Supplementary Table S3), conditions in which the most severe TA phenotypes were observed (Supplementary Table S1). MGDG and DGDG molecular species containing two TA, C-34:6 and C-36:6, were reduced in *2cpab* mutant leaves as compared to the WT (Fig. 4, C and D, Supplementary Table S3). Notably, the decrease in MGDG(C-34:6) in the *2cpab* mutant was accompanied by an increase in MGDG(C-34:5) and, to a greater extent, in MGDG(C-34:4), whereas the decrease in MGDG(C-36:6) was offset by the increase in the remaining MGDG molecular species (Fig. 4C). Therefore, although all MGDG molecular species with two TA were affected in the *2cpab* mutant, a marked effect was observed on prokaryotic MGDG, implying that the deficiency of 2-Cys Prxs might primarily affect the desaturation of 16:2 and 18:2 in the prokaryotic MGDG. In line with this notion, DGDG(C-36:6) and DGDG(C-34:6) were reduced in *2cpab* mutant leaves, and there was also a decrease in DGDG(C-34:3) (Fig. 4D), the most abundant DGDG molecular species from the prokaryotic pathway (Frentzen 1993; Wallis and Browse 2002). These data indicate that the deficiency of 2-Cys Prxs alters TA levels of all membrane lipids, with more prominent effects on ω-3 desaturation in the prokaryotic pathway, where FAD8 plays a substantial role synthesizing 18:3 in PG (McConn et al. 1994).

### The levels of 2-Cys Prxs correspond with the contents of FAD8

**Figure 5.**
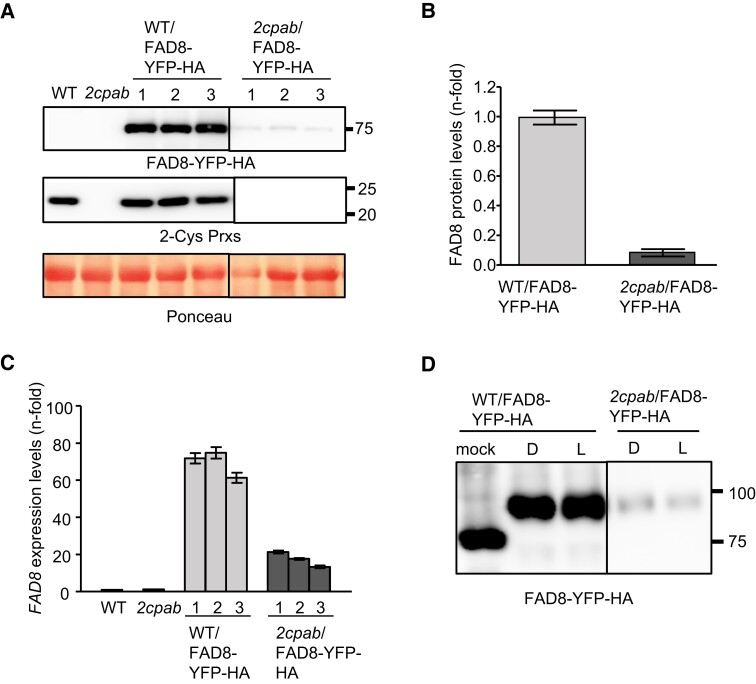
Effect of the deficit of 2-Cys Prxs on the expression and the redox state of FAD8 in rosette leaves from WT/FAD8–YFP–HA and *2cpab*/FAD8–YFP–HA transgenic plants. **A)** Western blot analysis of the levels of YFP–HA tagged Fatty Acid Desaturase 8 (FAD8) and 2-Cys peroxiredoxins (Prxs) in protein extracts (15 *µ*g) of rosette leaves from WT and *2cpab*, included as controls, and three individual (1, 2, and 3) from the WT/FAD8–YFP–HA and *2cpab*/FAD8–YFP–HA plants, grown under LD photoperiod for 4 wk. Even loading was monitored by Ponceau staining of the Rubisco large subunit. Molecular mass markers (kDa) are indicated on the right. **B)** Band intensities corresponding to FAD8–YFP–HA and Rubisco large subunit in WT/FAD8–YFP–HA and *2cpab*/FAD8–YFP–HA were quantified (GelAnalyzer). The contents of FAD8–YFP–HA in *2cpab*/FAD8–YFP–HA, normalized to the levels of Rubisco large subunit, are shown relative to the levels of the WT/FAD8–YFP–HA sample (arbitrarily assigned a value of 1). Data are given as the mean ± SEM. **C)** Levels of transcript of *FAD8* determined by RT-qPCR in the same individuals as in (**A**). Transcript levels were normalized against two reference genes (see Materials and Methods) and referenced against the levels in WT plants (arbitrarily considered as 1). Values represent the mean ± SEM of three technical replicates. **D)** In vivo redox state of YFP–HA tagged FAD8 in rosette leaves from WT/FAD8–YFP–HA and *2cpab*/FAD8–YFP–HA plants, grown as in (**A**), determined at the end of the dark period (**D**), and after 30 min of illumination at 150 *µ*E m^−2^ s^−1^ (L) by labeling of the thiol groups with the alkylating agent MM(PEG)_24_. A nonalkylated WT/FAD8–YFP–HA sample (mock) is shown as a control. Molecular mass markers (kDa) are indicated on the right.

Once established the direct link between 2-Cys Prxs and FAD8-mediated TA synthesis, we next assessed the functional relationship between both enzymes. First, we performed RT-qPCR expression analysis of the *FAD* genes, which showed no significant differences in the expression level of the *FAD8* gene in the *2cpab* mutant and the WT (Supplementary Fig. S5). Likewise, no significant differences between WT and the *2cpab* mutant were observed in the expression of additional ω-6 and ω-3 fatty acid desaturase genes, namely *FAD2*, *FAD3*, *FAD6*, and *FAD7* (Supplementary Fig. S5), indicating that 2-Cys Prxs-mediated regulation of fatty acid desaturation might occur by a posttranscriptional mechanism. To explore this possibility, we took advantage of a previously reported transgenic line (WT/FAD8–YFP–HA) overexpressing FAD8 fused to the YFP and HA tags in the WT background (Román et al. 2015). This line was manually crossed with the *2cpab* mutant and lines harboring the *FAD8*–*YFP*–*HA* construct in the *2cpab* background (*2cpab*/FAD8-YFP-HA) were selected among the progeny. Western blot analyses of protein extracts from leaves, probed with an anti-HA antibody, identified a band with the expected molecular weight (⁓75 kDa) corresponding to the FAD8–YFP–HA fusion protein in the WT/FAD8–YFP–HA line, which contained WT levels of 2-Cys Prxs (Fig. 5A). Interestingly, transgenic lines expressing the FAD8–YFP–HA fusion protein in the *2cpab* mutant, hence lacking 2-Cys Prxs, showed ∼10-fold decreased levels of FAD8, as compared to those in the WT/FAD8–YFP–HA line (Fig. 5, A and B). The decreased content of FAD8–YFP–HA in 2-Cys Prxs-deficient plants was accompanied by a ∼4-fold reduction in the expression levels of *FAD8* compared to those in WT/FAD8–YFP–HA plants (Fig. 5C). While the overexpression of *FAD8* was repressed to some extent in the *2cpab* background, these results suggest that 2-Cys Prxs affect FAD8 stability/abundance. Confocal microscopy analysis of FAD8–YFP–HA in the transgenic plants was also indicative of the association between the levels 2-Cys Prxs and the content of FAD8. In mesophyll cells of WT/FAD8–YFP–HA leaves, the YFP signal indicating the presence of FAD8 was detected in chloroplast envelopes and punctate spots that co-localized with chlorophyll autofluorescence (Supplementary Fig. S6), which is consistent with previous data (Román et al. 2015). However, fluorescence signals in leaves of the *2cpab*/FAD8–YFP–HA line were exclusively detected in envelopes from mesophyll chloroplasts, punctate spots being observed only in guard cells, suggesting differences in the mechanism(s) regulating FAD8 distribution in both cell types. The effect of 2-Cys Prxs on FAD8 protein stability/abundance, was further tested in additional transgenic lines generated by introducing the *FAD8* transgene, fused to the CFP and HA tags, into WT and *2cpab* plants (WT/FAD8–CFP–HA and *2cpab*/FAD8–CFP–HA, respectively). Three independent transgenic lines (#1, #2, and #3) in the WT and *2cpab* backgrounds, which accumulated different levels of the FAD8–CFP–HA protein (Supplementary Fig. S7, A and B), were selected for further analysis. Remarkably, transgenic lines #1 and #2 in each genetic background, overexpressing FAD8 transcripts to a similar extent (∼40-fold related to the WT), showed different FAD8 protein levels, being 2- to 3-fold higher in the WT than in the *2cpab* background. This trend, which was also observed in plants with weaker overexpression of FAD8 (lines #3, Supplementary Fig. S7, B and C), further confirms that 2-Cys Prxs regulate FAD8 at posttranscriptional level, influencing the protein stability and/or abundance in Arabidopsis leaves.

Arabidopsis FAD8, similarly to FAD7, contains five Cys residues located at positions 9, 102, 158, 274, and 284 (Gibson et al. 1994). The Cys-158 residue, conserved in Arabidopsis ω-6 and ω-3 fatty acid desaturases, forms part of the first His box (Los and Murata 1998), and appears to be essential for desaturase function, since its replacement by tyrosine resulted in a complete loss of FAD3 desaturase activity (McConn and Browse 1996; Román et al. 2015). Since it is well established the relevant function of 2-Cys Prxs in the control of the redox state of chloroplast proteins (Cejudo et al. 2021), a likely possibility is that the effect of the levels of 2-Cys Prxs on FAD8 is exerted via a redox mechanism. To test this possibility, we examined the in vivo redox state of FAD8 by thiol-specific labeling with the alkylating agent methylmaleimide polyethylene glycol (MM(PEG_24_)), in leaf protein extracts obtained from dark- and light-acclimated transgenic plants. Immunoblot analysis revealed a single band in WT/FAD8–YFP–HA protein extracts shifted in the presence of the alkylating agent (Fig. 5D), indicating labeling of Cys residue(s) in its thiolic form. The electrophoretic mobility of this shifted band was similar in the *2cpab*/FAD8–YFP–HA line, regardless the genetic background and light conditions, though the levels of FAD8 were almost depleted, indicating that the redox state of cysteines in FAD8 were not altered by the content of 2-Cys Prxs.

### 2-Cys Prxs and FAD8 are components of a large membrane protein complex

**Figure 6.**
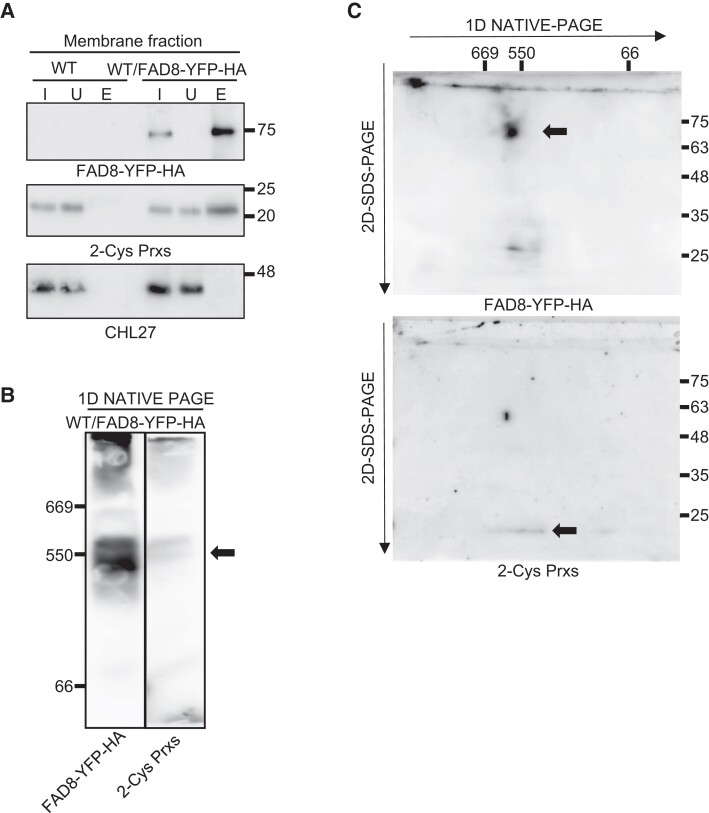
Interaction of 2-Cys Prxs and FAD8–YFP–HA in a large membrane protein complex. WT and transgenic plants overexpressing FAD8 fused to the YFP and HA tags in the WT background (WT/FAD8–YFP–HA) were grown under LD photoperiod for 4 wk. **A)** Membrane fractions obtained from formaldehyde-fixed leaves of WT and WT/FAD8–YFP–HA plants, were subjected to immunoprecipitation using anti-HA antibody coupled to magnetics beads. Proteins preparations (2 *µ*g) from the input (I) and unbounded (U) fractions and ∼25% of the total eluted volume (E) were immunoblotted and probed with anti-HA, indicating the presence of FAD8–YFP–HA, anti-2-Cys peroxiredoxins (Prxs), and anti-CHL27 (COPPER RESPONSE DEFECT 1). **B)** Membrane fraction proteins (40 *µ*g) from leaves of WT/FAD8–YFP–HA plants, were separated in 1D-NATIVE-PAGE (3% to 12% acrylamide), transferred to nitrocellulose filters and probed with anti-HA and anti-2-Cys Prxs antibodies. Molecular mass markers (kDa) are indicated (**A**, right; **B**, left). **C)** Membrane fraction proteins (40 *µ*g) from leaves of WT/FAD8–YFP–HA plants separated in 1D-NATIVE-PAGE as in **(B)** were subjected to 2D-SDS–PAGE (12% acrylamide) under reducing conditions, transferred to nitrocellulose filters and probed with anti-HA and anti-2-Cys Prxs antibodies. Molecular mass markers (kDa) are indicated on the top (nondenaturing) and on the right (denaturing). Arrows indicate the anti-HA and anti-2-Cys Prxs signals in 1D-NATIVE-PAGE (**B**) and 2D-SDS–PAGE (**C**).

FAD8 is localized in the Arabidopsis chloroplast envelope (Froehlich et al. 2003; Román et al. 2015). Likewise, although 2-Cys Prxs predominantly localize in chloroplast stroma (Peltier et al. 2006), its oligomeric form associates to thylakoid membranes (König et al. 2002; Muthuramalingam et al. 2009) and chloroplast envelope proteins (Liebthal et al. 2020). Hence, we hypothesized that 2-Cys Prxs may contribute to TA synthesis through stabilization or association with FAD8. If this were the case, both enzymes would interact either directly or indirectly, forming part of a protein complex. To explore this possibility, we performed biochemical fractionation of leaf protein extracts into soluble and membrane fractions, which were then subjected to Western blot analysis. As expected, 2-Cys Prxs were predominantly detected in the soluble fraction, however, though to a much lesser extent, it was also detected in membrane preparations (Supplementary Fig. S8). The fact that the large subunit of RUBISCO (RbcL) and COPPER RESPONSE DEFECT 1 (CHL27), a chloroplast membrane marker protein (Tottey et al. 2003), were detected in the soluble and membrane fractions, respectively, indicated the absence of contamination between the two preparations. Subsequently, interaction between 2-Cys Prxs and FAD8 was assessed by co-immunoprecipitation (Co-IP) assays with formaldehyde-fixed leaves of WT/FAD8–YFP–HA and WT plants, included here as a control. To this end, membrane fractions from both lines were immunoprecipitated with the anti-HA antibody coupled to magnetics beads. Immunoblotting indicated the high efficiency of the immunoprecipitation of FAD8–YFP–HA in membrane preparations as signal was detected in the input, enriched in the elution, and absent in the unbound fraction (Fig. 6A). Interestingly, a band corresponding to 2-Cys Prxs was also detected in the eluted fraction from membranes of WT/FAD8–YFP–HA, but not of WT plants (Fig. 6A), indicating the Co-IP of 2-Cys Prxs and FAD8. Moreover, CHL27, included here as a negative control, was not immunoprecipitated with the anti-HA antibody in membrane fractions of neither the WT/FAD8–YFP–HA nor the WT plants (Fig. 6A). The interaction of 2-Cys Prxs and FAD8 was additionally confirmed by Co-IP assays using the line WT/FAD8–CFP–HA#3, containing markedly lower levels of FAD8 than the WT/FAD8–YFP–HA line (Supplementary Fig. S9, A and B).

Once established that 2-Cys Prxs co-immunoprecipitated with FAD8 in Arabidopsis leaves, we examined whether both enzymes form part of the same protein complex(es) in vivo. To this end, membrane proteins from leaves of WT/FAD8–YFP–HA plants were separated by 1D-PAGE under native conditions and immunoblotted with anti-HA, indicating the presence of FAD8, and anti-2-Cys Prxs antibodies. Remarkably, both FAD8 and 2-Cys Prxs showed similar electrophoretic mobility in extracts from WT/FAD8–YFP–HA, in protein complexes with a molecular weight corresponding approximately to 550 kDa (Fig. 6B). The fact that 2-Cys Prxs were detected with a similar electrophoretic mobility (∼550 kDa) in extracts from the WT, but not from *2cpab* plants (Supplementary Fig. S10A), confirmed the specificity of the anti-2-Cys Prx antibody, similar to the anti-HA antibody, which did not show any detectable signal in WT extracts (Supplementary Fig. S10B). Proteins separated by 1D-PAGE were then subjected to denaturing SDS–PAGE in the second dimension. Immunoblots of 2D gels confirmed the presence of FAD8–YFP–HA, with a molecular weight of ∼75 kDa, and the monomer of 2-Cys Prxs, with the expected electrophoretic mobility below the 25 kDa marker, as part of the same macromolecular complex of ∼550 kDa in leaf membranes (Fig. 6C).

## Discussion

Photosynthetic performance, among other chloroplast processes, largely relies on redox-regulatory systems, which modulate enzyme activity based on reversible thiol–disulfide exchange reactions (Cejudo et al. 2021). Additionally, photosynthetic activity is adjusted in response to environmental cues by changes in chloroplast membrane lipid composition, in particular, the unsaturation balance of thylakoid lipids (Cook et al. 2021). In this work, we investigated the connection between chloroplast redox systems and lipid metabolism by focusing on the participation of 2-Cys Prxs, which play a central role in such posttranslational regulatory mechanism (Pérez-Ruiz et al. 2017). Our results demonstrate that desaturation of 16:2 and 18:2 fatty acids in chloroplast lipids, performed by ω-3 FADs, is compromised in Arabidopsis mutants deficient in 2-Cys Prxs (Fig. 1;Supplementary Table S1), hence raising the possibility that the synthesis of TA, 16:3 and 18:3, is linked to the chloroplast redox state through 2-Cys Prxs. Such effect does not occur at the transcriptional level (Supplementary Fig. S5), in agreement with previously reported transcriptomic data (Ojeda et al. 2021), in which ω-3 desaturase genes were not detected among the differentially expressed genes in the *2cpab* mutant. The finding that 2-Cys Prxs influence chloroplast TA levels in a dose-dependent manner (Fig. 1) appears to be contradictory with the fact that *2cpa* and *2cpb* single mutants lacking exclusively 2-Cys Prx A or 2-Cys Prx B, respectively, did not display remarkable differences in their fatty acid composition when compared with the WT (Supplementary Fig. S1; Horn et al. 2020). Nevertheless, the *2cpab* is a double knockout mutant devoid of both 2-Cys Prxs, A and B, present in Arabidopsis chloroplasts (Ojeda et al. 2018), whereas the *Δ2cp* mutant is a severe knockdown containing no 2-Cys Prx B and decreased levels (less than 5%) of 2-Cys Prx A (Pulido et al. 2010). Therefore, the 2-Cys Prxs content in *2cpa* and *2cpb* single mutants, previously established as 35% and 71%, respectively (Pérez-Ruiz et al. 2017), may be sufficient to ensure proper TA synthesis in Arabidopsis leaves.

A possible explanation for the altered TA contents in plants with severely reduced levels of 2-Cys Prxs is that these enzymes, as efficient thiol-dependent peroxidases able to reduce H_2_O_2_ and organic peroxides (König et al. 2002), exert a protective role against oxidation of chloroplast desaturases. In line with this possibility, it was reported that Prx Q is necessary for FAD4 desaturase activity, probably by preventing its oxidation (Horn et al. 2020). Similarly, H_2_O_2_ inhibits the activity of the soluble stearoyl-CoA desaturase while catalase stimulates its activity (Jeffcoat et al. 1978; McKeon and Stumpf 1982). However, HL-induced oxidative stress did not markedly affect TA levels in the *2cpab* mutant (Fig. 2A). Moreover, the deficiency of other chloroplast thiol-dependent peroxidases, namely Prx IIE and Prx Q, had negligible impact on fatty acid composition (Fig. 2B). However, it should be noted that the *prxq* mutant used here is a severe knockdown (Ojeda et al. 2018), whereas the *prxq* allele reported by Horn et al. (2020), which displayed decreased levels of 16:1t, is a knockout mutant, suggesting that low levels of Prx Q appear to be sufficient to produce WT levels of 16:1t. Altogether, our results indicate a more severe effect of 2-Cys Prxs deficiency on TA synthesis, which seems to be ROS-independent.

The fact that the *2cpab* mutant displayed impaired content of TA in the main thylakoid membranes lipids, i.e. MGDG, DGDG, and PG (Fig. 1, B and C and Supplementary Table S1), allows to conclude that 2-Cys Prxs affect chloroplast ω-3 FADs, i.e. FAD7 and FAD8. Nevertheless, the *2cpab* mutant, but not the *Δ2cp* mutant, also displays an increased 18:2/18:3 ratio in phospholipids such as phosphatidylinositol (PI), phosphatidylserine (PS), PC, phosphatidylethanolamine (PE), and PA, which are predominantly distributed in extrachloroplastic membranes. Similarly, lipidomic analysis revealed that although molecular species of prokaryotic origin were primarily affected by the deficiency of 2-Cys Prxs, galactolipids containing two TA from both biosynthetic pathways decreased in the *2cpab* mutant (Fig. 4, C and D). This could be explained by lipid exchange between cell compartments to alleviate the deficiency of ω-3 desaturation in chloroplasts completely devoid of 2-Cys Prxs, a compensation mechanism previously observed in *fad6* and *fad7* mutants (Browse et al. 1986; Miquel and Browse 1992; LaBrant et al. 2018), defective in plastidial ω-6 and ω-3 FADs, respectively. However, albeit to a lesser extent, an effect of 2-Cys Prxs on ω-3 fatty acid desaturation in the eukaryotic pathway cannot be ruled out. At optimal temperature for growth, the two chloroplast ω-3 desaturases perform nonredundant functions as FAD7 is mainly responsible for the desaturation of both 16:2 and 18:2 in galactolipids, MGDG and DGDG, whereas FAD8 is highly specific for 18:2 with a higher preference for PG (Browse et al. 1986; McConn et al. 1994; Román et al. 2015). The fact that 18:2/18:3 ratio in the *2cpab* mutant was like that of the *fad8* mutant, whereas 16:2/16:3 was higher than in the *fad8* mutant, but much lower than in *fad7* (Fig. 4, A and B), suggested that FAD8, and to a lesser extent FAD7, are functionally linked to 2-Cys Prxs. Interestingly, the effect of 2-Cys Prxs deficiency on prokaryotic MGDG was not as severe as the previously reported for the *fad7* mutant, in which the prokaryotic PG(C-34:4) was not altered in comparison to the WT (Bromke et al. 2015), further supporting the notion that 2-Cys Prxs exert a minor contribution to FAD7 function. These findings could be explained by a spatial organization of distinct PA pools to produce thylakoid membrane lipids through each glycerolipid biosynthetic pathway, similarly to the metabolically different pools of PC involved in the lipid trafficking out of and into the chloroplast for eukaryotic galactolipid biosynthesis (Karki et al. 2019).

In plants, the regulation of FAD8 activity in response to biotic and abiotic stresses allows the adjustment of membrane fluidity to temperature changes (Matsuda et al. 2005; Román et al. 2015) and jasmonic acid (JA)-mediated signaling (Soria-Garci et al. 2019). However, the molecular mechanism(s) underlying such regulation are still unknown. The tight association between the levels of 2-Cys Prxs and the accumulation of 18:3-PG (Fig. 1C and Supplementary Table S1), which is preferentially formed by FAD8 activity, together with the fact that the expression of the genes encoding ω-6 and ω-3 FADs in Arabidopsis was not altered in the *2cpab* mutant (Supplementary Fig. S5), suggest that 2-Cys Prxs affect FAD8 function posttranscriptionally. Because antibodies against FAD8 are not available, we tested this possibility in transgenic lines overexpressing FAD8 tagged with YFP–HA (Fig. 5A) or CFP–HA (Supplementary Fig. S7A) in the WT and *2cpab* mutant backgrounds. Remarkably, the accumulation of FAD8 protein, regardless of transcript levels, positively associated with the presence of 2-Cys Prxs (Fig. 5, B and C and Supplementary Fig. S7, B and C), supporting the notion that the desaturase function of FAD8 is mainly posttranslationally modulated by 2-Cys Prxs. Such regulatory role of 2-Cys Prxs on TA synthesis, however, is not exerted by cysteine-based redox exchange reactions in FAD8, as shown by the alkylation labeling of the thiol groups of the enzyme (Fig. 5D).

The redox balance of 2-Cys Prxs mainly depends on NTRC, which plays a crucial role in chloroplast function (Pérez-Ruiz et al. 2017). Both enzymes form a redox relay that adjusts the redox state of chloroplast enzymes in response to light (Cejudo et al. 2021). Perturbations of 2-Cys Prxs redox balance, either by the lack (Ojeda et al. 2021) or the overexpression of NTRC (Ojeda et al. 2018) impair the reductive and oxidative regulation of chloroplast enzymes. Conversely, DA/TA ratios were not altered in Arabidopsis leaves from the *ntrc* mutant or NTRC-OE plants (Fig. 3, A to C) indicating that the function of 2-Cys Prxs on thylakoid fatty acid unsaturation is exerted beyond the classic redox regulatory mechanism of plant chloroplasts, hence resembling the recently reported function of these enzymes in chloroplast differentiation during embryogenesis (Gallardo-Martínez et al. 2023). Nevertheless, a mutant variant of 2-Cys Prx A in which the C_P_ residue at the active site of the enzyme was replaced by Ser failed to complement the TA phenotype of the *2cpab* mutant (Supplementary Fig. S2, B and C), indicating that this function of 2-Cys Prxs requires the redox active form of the enzyme. In addition to its well-known peroxidase activity, 2-Cys Prxs exhibit a still poorly understood molecular chaperone function, which appears to depend on the oligomeric conformation of the enzyme, strongly influenced by the redox state of the C_P_ and C_R_ residues (Kim et al. 2009; König et al. 2013; Puerto-Galán et al. 2013). In leaves, aggregates of 2-Cys Prxs attach to thylakoid membranes of barley (König et al. 2002) and Arabidopsis (Muthuramalingam et al. 2009), this association being enhanced under oxidizing conditions. Accordingly, a minor fraction of 2-Cys Prxs was detected in membrane preparations from leaves of Arabidopsis (Supplementary Fig. S8) and, remarkably, the enzyme co-immunoprecipitates with FAD8 (Fig. 6A and Supplementary Fig. S9B). For this interaction to occur, either directly or indirectly, 2-Cys Prxs should be located in close proximity to the inner envelope membrane, where FAD8 is localized (Froehlich et al. 2003; Román et al. 2015). In support of this notion, an approach based on blue-native PAGE and tandem mass spectrometry identified 2-Cys Prx B in high molecular weight protein complexes from chloroplast envelopes (Takabayashi et al. 2017). Likewise, Co-IP of RHOMBOID-LIKE PROTEIN 10 (RBL10), an inner envelope membrane protein, yielded 2-Cys Prx A among the putative interactors (Lavell et al. 2021). In addition, 2-Cys Prxs have been proposed to interact with a supramolecular complex in the inner membrane of chloroplast envelope comprising lipoxygenase 2 (LOX2), allene oxide synthase (AOS), and allene oxide cyclase (AOC) (Liebthal et al. 2020), which is involved in substrate channeling for JA biosynthesis (Pollmann et al. 2019). Consistent with these findings, 2-Cys Prxs and FAD8 were detected as components of a large membrane protein complex of ∼550 kDa (Fig. 6, B and C). Given that FAD6/FAD7 and FAD6/FAD8 form heterodimers that might facilitate metabolic channeling of glycerolipids to produce thylakoid membrane lipids in Arabidopsis (Lou et al. 2014; Soria-García et al. 2019) and that TA are the main substrates of LOX (Wasternack and Feussner 2018), it is tempting to speculate that 2-Cys Prxs, FAD6/FAD8, and LOX2/AOS/AOC are components of the same complex. The association of 2-Cys Prxs with these inner envelope membrane embedded complexes is compatible with their possible function as scaffold/chaperone proteins, promoting the stabilization or assembly of macromolecular complexes containing FAD8, and thus, the synthesis of TA in PG, a membrane glycerolipid mostly formed via the prokaryotic pathway. It is well established that PG levels and fatty acid composition play an important role in plant thermotolerance and photosynthetic performance (Wada and Murata 2007; Kobayashi et al. 2016; Hernández and Cejudo 2021; Fujii et al. 2022; Hoh et al. 2022). Therefore, besides the central function of 2-Cys Prxs in chloroplast redox homeostasis (Cejudo et al. 2021), these enzymes may also participate in plant response to environmental cues by modulating lipid composition of thylakoid membranes, though further investigations are needed to elucidate the molecular mechanism underlying this function of 2-Cys Prxs.

## Materials and methods

### Biological material and growth conditions

Arabidopsis (*A. thaliana*) WT, ecotype Columbia-0, T-DNA insertion mutants, and transgenic lines (Supplementary Table S4) were routinely grown on soil in growth chambers under long-day (LD) (16 h light/8 h darkness) or SD (8 h light/16 h darkness) photoperiod at 22 and 20 °C during light and dark periods, respectively, and a light intensity of 125 *µ*E m^−2^ s^−1^ unless otherwise stated. For HL conditions, 6-wk-old SD grown plants were transferred to continuous HL (930 *µ*E m^−2^ s^−1^) for 7 d. T-DNA insertion mutants *2cpab* (Ojeda et al. 2018), *Δ2cp* (Pulido et al. 2010), *ntrc* (Serrato et al. 2004), *prxQ* (Lamkemeyer et al. 2006), *prxIIE* (Romero-Puertas et al. 2007), and *fad7* and *fad8* (Román et al. 2015) were previously reported. Transgenic lines overexpressing 2CPA (2CPA-OE), NTRC (NTRC-OE) or FAD8 (WT/FAD8–YFP–HA) under the control of the CaMV 35S promoter in the Col-0 background, were previously described by Pérez-Ruiz et al. (2017), Ojeda et al. (2018), and Román et al. (2015), respectively. Transgenic lines *2cpab/2CPA-OE*, *2cpab/2CPA-C_P_-S-OE*, *2cpab*/FAD8–YFP–HA, WT/FAD8–CFP–HA, and *2cpab*/FAD8–CFP–HA were generated in this study as described below. *Escherichia coli* and *Agrobacterium tumefaciens* (strain GV3101) were grown in liquid Miller nutrient at 37 and 28 °C, respectively, with the appropriate antibiotics.

### Vector construction and generation of transgenic plants

For the generation of *2cpab/2CPA-OE* and *2cpab/2CPA-C_P_-S-OE* transgenic lines, cDNA was synthesized with the Maxima first strand cDNA synthesis kit (Thermo Scientific) from total RNA isolated from WT Arabidopsis leaves using Trizol reagent (Invitrogen). The cDNA sequence coding for 2-Cys PRX A, including the stop codon, was amplified with iProof High-Fidelity DNA Polymerase (Bio-Rad) using specific oligonucleotides (Supplementary Table S5) that added attB recombination sites at the 5′ and 3′ ends, respectively. The PCR product was cloned in the Gateway compatible vector pDONR221 (Invitrogen) and confirmed by sequencing. The *2CPA-C_P_-S* mutant version, in which the peroxidatic Cys (C_P_) of 2-Cys Prx A was replaced by Ser (C119S: codon change TGC > AGC), was generated by site-directed mutagenesis as reported in (Pérez-Ruiz et al. 2006), using the pDONR221/2CPA plasmid and specific oligonucleotides (Supplementary Table S5). Plasmids pDONR221/2CPA and pDONR221/2CPA-C_P_-S were then subcloned into the binary vector pEARLEYGATE100 using LR clonase (Invitrogen) according to the manufacturer´s instructions. Constructed vectors were introduced into *A. tumefaciens* (GV3101) and transformed into the *2cpab* mutant by the floral dipping method (Clough and Bent 1998). For the generation of *2cpab*/FAD8–YFP–HA transgenic line, the WT/FAD8–YFP–HA line (Román et al. 2015), which harbors the BASTA resistance, was manually crossed with the *2cpab* double mutant. Plants resulting from this cross were checked for heterozygosity of the T-DNA insertions in the *2-Cys PRX A* and *2-Cys PRX B* genes and self-pollinized. The *2cpab*/FAD8–YFP–HA line was identified in the progeny based on the BASTA resistance and by PCR analysis of genomic DNA with oligonucleotides listed in Supplementary Table S5. For the generation of WT/FAD8–CFP–HA and *2cpab*/FAD8–CFP–HA transgenic lines, the genomic fragment of *FAD8* excluding the stop codon (2,012 bp) was amplified with iProof High-Fidelity DNA Polymerase (Bio-Rad) using specific oligonucleotides (Supplementary Table S5) that added attB recombination sites at the 5′ and 3′ ends, respectively. The PCR product was cloned in the Gateway compatible vector pDONR221 (Invitrogen) and confirmed by sequencing. Resulting plasmid pDONR221/FAD8 was then subcloned, as indicated above, into the binary vector pEARLEYGATE102, which add CFP and HA C-terminal tags. Constructed vector was introduced into *A. tumefaciens* (GV3101) and transformed into the WT and *2cpab* genetic backgrounds as described above.

### Protein extraction, western blot analysis and alkylation assays

Plant tissues, frozen in liquid nitrogen, were ground to a fine powder using mortar and pestle. For total protein analysis, extraction buffer A (50 mM Tris–HCl, pH 8.0, 0.15 M NaCl, 0.5% (v/v) Nonidet P-40) was immediately added, mixed on a vortex, and centrifuged at 16,100 × *g* at 4 °C for 20 min. For FAD8–YFP–HA protein analysis, powdered samples were resuspended in 50 mM Tris–HCl, pH 7.8, 2.5% (vol/vol) glycerol, 4 M urea, and 2% SDS. For the separation of soluble and membrane protein fractions, the powder was resuspended in buffer B (25 mM HEPES–KOH, pH 7.4, 10 mM MgCl_2_, 0.2 mM phenylmethanesulfonyl fluoride, 5 mM aminocaproic acid) and filtered through a layer of Miracloth paper (Calbiochem, Darmstadt, Germany). After centrifugation, 6,000 × *g* at 4 °C for 5 min, the supernatant was collected as the soluble fraction and the pellet containing the membrane proteins was solubilized in buffer C (50 mM Tris–HCl, pH 8, 0.15 M NaCl, 1% (w/v) digitonin, 1% (w/v) *n*-dodecyl-β-D-maltoside), incubated on ice for 10 min and centrifuged at 13,500 *× g* at 4 °C for 10 min. After centrifugation, supernatant was collected as the membrane fraction. Protein samples were quantified using the Bradford reagent (Bio-Rad). Protein extracts were subjected to SDS–PAGE under reducing (NTRC, 2-Cys Prxs, FAD8-YFP-HA, CHL27) or nonreducing (2-Cys Prxs) conditions using acrylamide gel concentration of 12%. For 1D-NATIVE-PAGE, membrane proteins were separated by nondenaturing electrophoresis (3% to 12% or 5% to 12% acrylamide gradient) at 4 °C and at 30 V overnight. For 2D-SDS–PAGE, resolving proteins by native electrophoresis was followed by separation in a second dimension with denaturing SDS–PAGE (12% acrylamide). Proteins resolved under denaturing or nondenaturing conditions, as indicated, were transferred to nitrocellulose membranes and probed with specific antibodies for NTRC (Serrato et al. 2004), 2-Cys Prxs (Pérez-Ruiz et al. 2006), HA, and CHL27 (Agrisera). Thiol labeling assay by the alkylating agent MM(PEG)_24_ was performed as previously described (Pérez-Ruiz et al. 2017).

### Lipid analysis

Total lipids were extracted from Arabidopsis leaves using the chloroform/methanol method described by Li-Beisson et al. (2013), and lipids separation was carried out by thin-layer chromatography as described (Hernández et al. 2008). Fatty acid methyl esters of total lipids or individual lipid classes were produced by acid-catalyzed transmethylation (Garcés and Mancha 1993) and analyzed by gas chromatography using a GC-MS-QP2010 Plus (Shimadzu, Kyoto, Japan) fitted with a Suprawax 280 capillary column (10 m length, 0.1 mm i.d., and 0.1 µm film thickness) and one quadrupole mass detector. Helium was used as the carrier gas at a flow rate of 16.3 mL/min. Ion source was held at a temperature of 200 °C, and an interphase temperature of 280 °C. Heptadecanoic acid was used as internal standard for lipid content calculations. Lipidomic analyses were performed by the Analytical Laboratory of the Kansas Lipidomics Research Center (KLRC) at Kansas State University. The profiles of galactolipid molecular species were measured by an automated electrospray ionization–tandem mass spectrometry methods described by Welti et al. (2002).

### RT-qPCR

Quantitative PCR (qPCR) was performed using cDNA synthesized as stated above in a IQ2 real-time PCR detection system (Bio-Rad) as previously reported (Pérez-Ruiz et al. 2017). Oligonucleotides used for qPCR analyses are listed in Supplementary Table S5. Expression levels were normalized using *ACTIN2* and *UBQ10* as reference genes.

### Confocal microscopy analysis

Subcellular localization of FAD8 was analyzed by confocal laser scanning microscopy (FLUOVIEW FV3000, Olympus) of leaves dissected from WT/FAD8–YFP–HA and *2cpab*/FAD8–YFP–HA transgenic plants, expressing the corresponding FAD8–YFP–HA fusion protein. Images were acquired with a UPlanXApo 40x/1.4 NA oil immersion objective. Samples were excited with 488 nm laser at 1% of intensity, and fluorescence emission was collected across windows of 520 to 570 nm on high-sensitivity spectral detectors at 450 V, 1× of gain and 4% of offset for YFP and 650 to 750 nm and at 360–430 V, 1× of gain and 4% of offset for chlorophyll autofluorescence. Images were analyzed using Olympus FV31S-SW (Olympus Corporation) and ImageJ software (National Institute of Health, USA).

### Immunoprecipitation

Rosette leaves from WT/FAD8–YFP–HA, WT/FAD8–CFP–HA#3, and WT plants, used as a negative control, were fixed with 1% (v/v) formaldehyde by vacuum infiltration for 35 min. Cross-linking reaction was stopped by adding 300 mM glycine followed by three washes with Milli-Q water. Membrane protein fractions (∼840 *µ*g), obtained as described above, were incubated with 30 *µ*L HA magnetic beads (Invitrogen) at 4 °C for 1.5 h on a rotating wheel. The beads were washed three times with 50 mM Tris–HCl, pH 7.5, 150 mM NaCl, 5% (v/v) glycerol, and 0.05% (v/v) Nonidet P40 and then two times with 50 mM Tris–HCl, pH 7.5, 150 mM NaCl and 5% (v/v) glycerol. Proteins were eluted from the beads by 10 min boiling in 100 *µ*L SDS–PAGE loading buffer.

### Accession numbers

Sequence data from this article can be found in the GenBank/EMBL data libraries under accession numbers AY079107 (2-Cys Prx A) and AY078043 (FAD8).

## Supplementary Material

kiae102_Supplementary_Data

## Data Availability

All data that support the findings in this paper are available within the article and its Supporting Information or are available from the corresponding authors upon reasonable request.

## Supplementary data

The following materials are available in the online version of this article.

**Supplementary Table S1.** Dienoic to trienoic fatty acids (DA/TA) ratios from leaves of *2cpab* mutant plants grown under LD and SD conditions.

**Supplementary Table S2.** Fatty acid composition and DA/TA ratios of WT and *2cpab* mutant leaves after 1 wk at control (SD, 120 *µ*E m^−2^ s^−1^) or HL (continuous, 930 *µ*E m^−2^ s^−1^) conditions.

**Supplementary Table S3.** Lipidomic analysis of galactolipids molecular species (nmol/mg DW) in rosette leaves from WT and *2cpab* mutant plants grown under SD photoperiod.

**Supplementary Table S4.** Arabidopsis mutants and transgenic lines used in this study.

**Supplementary Table S5.** Arabidopsis oligonucleotides used in this study.

**Supplementary Figure S1.** Fatty acid composition of Arabidopsis leaves from WT and 2-Cys Prxs deficient mutants.

**Supplementary Figure S2.** DA/TA ratios in *2cpab*/2CPA-OE and *2cpab*/2CPA-C_P_-S transgenic lines.

**Supplementary Figure S3.** Polar lipid composition of Arabidopsis rosette leaves from WT and 2-Cys Prxs deficient mutant plants.

**Supplementary Figure S4.** Effect of the overexpression of 2-Cys Prx A on TA levels in Arabidopsis leaves.

**Supplementary Figure S5.** RT-qPCR expression analysis of ω-6 and ω-3 desaturases in WT and the *2cpab* mutant.

**Supplementary Figure S6.** Subcellular localization of FAD8 in rosette leaves from WT/FAD8–YFP–HA and *2cpab*/FAD8–YFP–HA plants.

**Supplementary Figure S7.** Effect of the deficit of 2-Cys Prxs on the expression of FAD8 in rosette leaves from WT/FAD8–CFP–HA and *2cpab*/FAD8–CFP–HA transgenic plants.

**Supplementary Figure S8.** Accumulation of 2-Cys Prxs in soluble and membrane protein fractions from Arabidopsis leaves.

**Supplementary Figure S9.** Interaction of 2-Cys Prxs and FAD8–CFP–HA in Arabidopsis leaf membranes.

**Supplementary Figure S10.** Validation of the specificity of antibodies against 2-Cys Prxs and the HA-tag in Western blot analysis of native proteins.

## Dive Curated Terms

The following phenotypic, genotypic, and functional terms are of significance to the work described in this paper:

FAD3 Gramene: AT2G29980
FAD3 Araport: AT2G29980

FAD6 Gramene: AT4G30950
FAD6 Araport: AT4G30950

aos Gramene: AT5G42650
aos Araport: AT5G42650

LOX2 Gramene: AT3G45140
LOX2 Araport: AT3G45140

SDS Gramene: AT1G14750
SDS Araport: AT1G14750

FAD8 Gramene: AT5G05580
FAD8 Araport: AT5G05580

UBQ10 Gramene: At4g05320
UBQ10 Araport: At4g05320

FAD7 Gramene: NCBI Gene ID: 544203
FAD7 Araport: NCBI Gene ID: 544203

FAD2 Gramene: NCBI GeneID: 820387
FAD2 Araport: NCBI GeneID: 820387

NTRC Gramene: AT2G41680
NTRC Araport: AT2G41680
