# Supplementary material for: 2-Cys peroxiredoxins contribute to thylakoid lipid unsaturation by affecting ω-3 fatty acid desaturase 8
Source: Plant Physiol. 2024 Feb 22;195(2):1521–35. doi: 10.1093/plphys/kiae102 (PMC11142380; doi:10.1093/plphys/kiae102)
Supplement: kiae102_Supplementary_Data [file kiae102_supplementary_data.pdf]

Supplemental Information for Hernández et al.

Supplemental Table S1. Dienoic to trienoic fatty acids (DA/TA) ratios from leaves of *2cpab* mutant plants grown under long-day (LD) and short-day (SD) conditions. Data are mean ± SD from three independent plants. ND, not detected. *Indicates significantly different to long-day photoperiod according to two-way ANOVA with a Bonferroni post-test ($p < 0.05$).

Lipid class	Photoperiod	DA/TA ratio	
		16:2/16:3	18:2/18:3
PI	LD	ND	2.0 ± 0.1
	SD	ND	2.1 ± 0.1
PS	LD	ND	2.0 ± 0.4
	SD	ND	2.2 ± 0.1
PC	LD	ND	1.9 ± 0.1
	SD	ND	2.0 ± 0.1
PE	LD	ND	2.8 ± 0.2
	SD	ND	2.5 ± 0.1
PA	LD	ND	2.0 ± 0.1
	SD	ND	2.1 ± 0.2
PG	LD	ND	1.7 ± 0.1
	SD	ND	2.4 ± 0.2*
MGDG	LD	0.4 ± 0.0	0.4 ± 0.0
	SD	0.7 ± 0.1*	0.6 ± 0.1
DGDG	LD	1.0 ± 0.1	0.5 ± 0.0
	SD	1.8 ± 0.2*	0.6 ± 0.0
SQDG	LD	ND	1.9 ± 0.2
	SD	ND	1.7 ± 0.2

DGDG, digalactosyldiacylglycerol; MGDG, monogalactosyldiacylglycerol; PA, phosphatidate; PC, phosphatidylcholine; PE, phosphatidylethanolamine; PI, phosphatidylinositol; PS, phosphatidylserine; SQDG, sulfoquinovosyldiacylglycerol.

Supplemental Table S2. Fatty acid composition and dienoic to trienoic fatty acid ratios of wild-type (WT) and *2cpab* mutant leaves after 1 week at control (short-day, 120 µE m^{-2} s^{-1}) or high-light (HL, continuous, 930 µE m^{-2} s^{-1}) conditions. Data are means ± SD of three independent plants. *Indicates significantly different to control condition according to two-way ANOVA with a Bonferroni post-test ($p < 0.05$).

Fatty acid	WT		*2cpab*	
	Control	HL	Control	HL
16:0	24.3 ± 1.2	27.5 ± 1.5*	23.9 ± 2.9	27.6 ± 2.4*
16:1c	0.6 ± 0.1	0.3 ± 0.1	0.8 ± 0.1	0.2 ± 0.2
16:1t	2.5 ± 0.1	0.9 ± 0.3	2.3 ± 0.2	1.0 ± 0.3
16:2	0.2 ± 0.0	0.2 ± 0.2	2.9 ± 0.7	1.4 ± 0.1
16:3	5.8 ± 0.9	1.9 ± 0.4*	3.2 ± 0.8	1.0 ± 0.1
18:0	8.5 ± 0.9	6.6 ± 0.7	8.6 ± 1.1	7.8 ± 1.2
18:1	2.3 ± 0.2	4.3 ± 0.2	4.3 ± 0.7	5.2 ± 1.2
18:2	17.4 ± 0.6	26.0 ± 1.3*	28.4 ± 0.4	36.7 ± 1.0*
18:3	38.4 ± 2.0	32.3 ± 2.5*	25.6 ± 2.4	19.0 ± 1.5*
16:2/16:3	0.04 ± 0.01	0.14 ± 0.05*	0.90 ± 0.01	1.34 ± 0.15*
18:2/18:3	0.45 ± 0.04	0.81 ± 0.10*	1.12 ± 0.12	1.94 ± 0.14*

16:0, palmitic acid; 16:1c, Δ9cis palmitoleic acid; 16:1t, Δ3trans palmitoleic acid, 16:2, palmitolinoleic acid; 18:0, stearic acid; 18:1, oleic acid; 18:2, linoleic acid; 18:3, linolenic acid.

Supplemental Table S3. Lipidomic analysis of galactolipids molecular species (nmol/mg DW) in rosette leaves from wild-type and *2cpab* mutant plants grown under short-day photoperiod. Data are means ± SD of five independent plants.

Mass	Compound Formula	Compound Name	WT		*2cpab*	
			Average	SD	Average	SD
764.5	$C_{43}H_{70}O_{10}$	MGDG(34:6)	20.56	2.08	11.58	1.03
766.5	$C_{43}H_{72}O_{10}$	MGDG(34:5)	2.60	0.49	4.84	0.61
768.5	$C_{43}H_{74}O_{10}$	MGDG(34:4)	0.61	0.32	3.06	0.66
770.5	$C_{43}H_{76}O_{10}$	MGDG(34:3)	0.50	0.11	0.58	0.19
772.6	$C_{43}H_{78}O_{10}$	MGDG(34:2)	0.12	0.05	0.17	0.08
774.6	$C_{43}H_{80}O_{10}$	MGDG(34:1)	0.09	0.03	0.12	0.04
792.5	$C_{45}H_{74}O_{10}$	MGDG(36:6)	10.57	2.06	6.34	1.01
794.5	$C_{45}H_{76}O_{10}$	MGDG(36:5)	0.91	0.05	2.20	0.20
796.6	$C_{45}H_{78}O_{10}$	MGDG(36:4)	0.38	0.11	0.93	0.15
798.6	$C_{45}H_{80}O_{10}$	MGDG(36:3)	0.06	0.02	0.11	0.04
800.6	$C_{45}H_{82}O_{10}$	MGDG(36:2)	0.01	0.00	0.02	0.01
802.6	$C_{45}H_{84}O_{10}$	MGDG(36:1)	0.01	0.00	0.01	0.00
820.6	$C_{47}H_{78}O_{10}$	MGDG(38:6)	0.85	0.45	0.63	0.22
822.6	$C_{47}H_{80}O_{10}$	MGDG(38:5)	0.04	0.01	0.03	0.01
824.6	$C_{47}H_{82}O_{10}$	MGDG(38:4)	0.08	0.03	0.07	0.01
		Total MGDG	37.41	5.37	30.71	3.46
926.6	$C_{49}H_{80}O_{15}$	DGDG(34:6)	0.63	0.07	0.38	0.04
928.6	$C_{49}H_{82}O_{15}$	DGDG(34:5)	0.20	0.04	0.32	0.05
930.6	$C_{49}H_{84}O_{15}$	DGDG(34:4)	0.09	0.03	0.27	0.03
932.6	$C_{49}H_{86}O_{15}$	DGDG(34:3)	2.23	0.14	1.15	0.12
934.6	$C_{49}H_{88}O_{15}$	DGDG(34:2)	0.31	0.03	0.63	0.07
936.6	$C_{49}H_{90}O_{15}$	DGDG(34:1)	0.20	0.07	0.26	0.05
954.6	$C_{51}H_{84}O_{15}$	DGDG(36:6)	6.82	0.46	4.18	0.39
956.6	$C_{51}H_{86}O_{15}$	DGDG(36:5)	0.47	0.05	2.21	0.25
958.6	$C_{51}H_{88}O_{15}$	DGDG(36:4)	0.15	0.02	0.62	0.07
960.6	$C_{51}H_{90}O_{15}$	DGDG(36:3)	0.14	0.02	0.18	0.01
962.6	$C_{51}H_{92}O_{15}$	DGDG(36:2)	0.02	0.01	0.04	0.01
964.7	$C_{51}H_{94}O_{15}$	DGDG(36:1)	0.01	0.00	0.01	0.01
982.6	$C_{53}H_{88}O_{15}$	DGDG(38:6)	0.32	0.15	0.31	0.11
984.6	$C_{53}H_{90}O_{15}$	DGDG(38:5)	0.03	0.00	0.03	0.00
986.6	$C_{53}H_{92}O_{15}$	DGDG(38:4)	0.03	0.00	0.04	0.01
988.7	$C_{53}H_{94}O_{15}$	DGDG(38:3)	0.00	0.00	0.01	0.00
		Total DGDG	11.66	0.94	10.64	0.96
		Total GL	49.07	6.29	41.35	4.04

GL, galactolipids; MGDG, monogalactosyldiacylglycerol; DGDG, digalactosyldiacylglycerol.

Supplemental Table S4. Arabidopsis mutants and transgenic lines used in this study.

Mutant/transgenic line	T-DNA line/ background	Protein	KO/KD/OE	Reference
2cpa	GABI_295C05	2-Cys Prx A	KO	Pérez-Ruiz *et al.* (2017)
2cpb	SALK_017213	2-Cys Prx B	KO	Kirchsteiger *et al.* (2009)
Δ*2cp*	SALK_065264<br>SALK_017213	2-Cys Prx A<br>2-Cys Prx B	KD<br>KO	Pulido *et al.* (2010)
2cpab	GABI_295C05<br>SALK_017213	2-Cys Prx A<br>2-Cys Prx B	KO<br>KO	Ojeda *et al.* (2018)
prxIIE	SALK_064512	Prx IIE	KD	Romero-Puertas *et al.* (2007)
prxQ	GABI_089H10	Prx Q	KD	Lamkemeyer *et al.* (2006)
ntrc	SALK_012208	NTRC	KO	Serrato *et al.* (2004)
fad7	SALK_147096C	FAD7	KO	Roman *et al.* (2015)
fad8	SALK_093590	FAD8	KO	Roman *et al.* (2015)
2cpab/2CPA-OE	*2cpab*	2-Cys Prx A	OE	This study
2cpab/2CPA-C_P-S-OE	*2cpab*	2-Cys Prx A-Cys119Ser	OE	This study
NTRC-OE	Wild-type	NTRC	OE	Ojeda et al. (2018)
2CPA-OE	Wild-type	2-Cys Prx A	OE	Perez-Ruiz *et al.* (2017)
WT/FAD8-YFP-HA	Wild-type	FAD8-YFP-HA	OE	Roman *et al.* (2015)
2cpab/FAD8-YFP-HA	*2cpab*	FAD8-YFP-HA	OE	This study
WT/FAD8-CFP-HA	Wild-type	FAD8-CFP-HA	OE	This study
2cpab/FAD8-CFP-HA	*2cpab*	FAD8-CFP-HA	OE	This study

KO, knockout line; KD, knockdown line; OE, overexpression line

Supplemental Table S5. Arabidopsis oligonucleotides used in this study.

**Genotyping primers**	**Locus**	**Sequence**
SALK T-DNA left border	T-DNA	5′-ATTTTGCCGATTTCGGAAC-3′
GABI T-DNA left border	T-DNA	5′-ATATTGACCATCATACTCATTGC-3′
2cpa (GABI_295C05)	At3g11630	5′-CTTCCACTGGTTGGAAACAAG-3′<br>5′-AATGCCTGCAACATTGAAAAC-3′
2cpb (SALK_017213)	At5g06290	5′-CCACCTGAACCAAGAAAG-3′<br>5′-CCTGCAAGACAACATCAC-3′
**Cloning primers**	**Locus**	**Sequence**
2-Cys Prx A (cDNA)	At3g11630	5′-AAAAAGCAGGCTtcATGGCGTCTGTTGCTTCT-3′<br>5′AGAAAGCTGGGTcCTAAATAGCTGAGAAGTACTC-3′
FAD8 (gDNA)	At3g11630	5′-AAAAAGCAGGCTtcATGGCGAGCTCGGTTTTA-3′<br>5′AGAAAGCTGGGTcTGTTCTTTGTCCATTGAG-3′
**Mutagenesis primers**	**Locus**	**Sequence**
2-Cys Prx A-C119S (cDNA)	At3g11630	5′-ACTTTACTTTCGTC**A**GCCCAACAGAGATT-3′<br>5′-AATCTCTGTTGGGC**T**GACGAAAGTAAAGT-3′
**qRT-PCR primers**	**Locus**	**Sequence**
FAD2	At3g12120	5′-AAGTATAGTCATCGCCGTCAC-3′<br>5′-ATTGCTGATTTCTGCTTTGGG-3′
FAD3	At2g29980	5′-CAACATTCATCACGACATTGGA-3′<br>5′-TGAGGGATTTGTGGGAAGAG-3′
FAD6	At4g30950	5′-ATGAGTCTATACAAGAGAACTGGG-3′<br>5′-CATTATCGTCTTCATCAATCGCC-3′
FAD7	At3g11170	5′-ATGGGTTCACGAGGAATTGG-3′<br>5′-GTGTCGTTAATGGTGTGCTC-3′
FAD8	At5g05580	5′-TTGCCTCTAACCCTAAACCC-3′<br>5′-GGGAATTGAGAAGAGAAGAAGGA-3′
ACTIN2	At3g18780	5′-GCACTTGCACCAAGCAGCAT-3′<br>5′-CCTTTCAGGTGGTGCAACGAC-3′
UBQ10	At4g05320	5′-GGCCTTGTATAATCCCTGATGAATAAG-3′<br>5′-AAAGAGATAACAGGAACGGAAACATAGT-3′

The attB recombination sites are underlined. Lower case letters indicate additional nucleotides included to maintain the proper reading frame. Mutated nucleotide is indicated by bold letter.

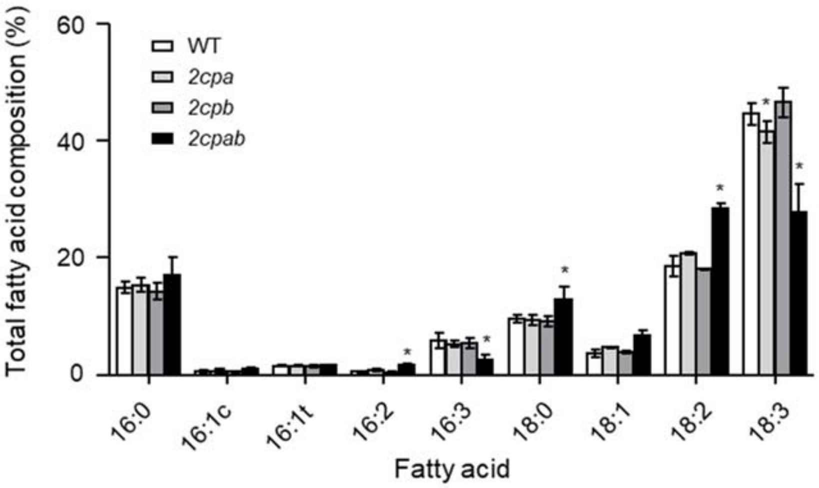

Supplemental Figure S1. Fatty acid composition of Arabidopsis leaves from wild-type and 2-Cys Prxs deficient mutants. Fatty acid composition of total lipids of rosette leaves from wild-type (WT) and the *2cpa*, *2cpb* and *2cpab* mutant plants grown under long-day conditions for 4 weeks. Total leaf lipids were extracted, and fatty acid composition analysed as described in Material and Methods. Data are means ± SD of four independent plants. Asterisks indicate significantly different ($p < 0.05$) to WT according to two-way ANOVA with a Bonferroni post-test.

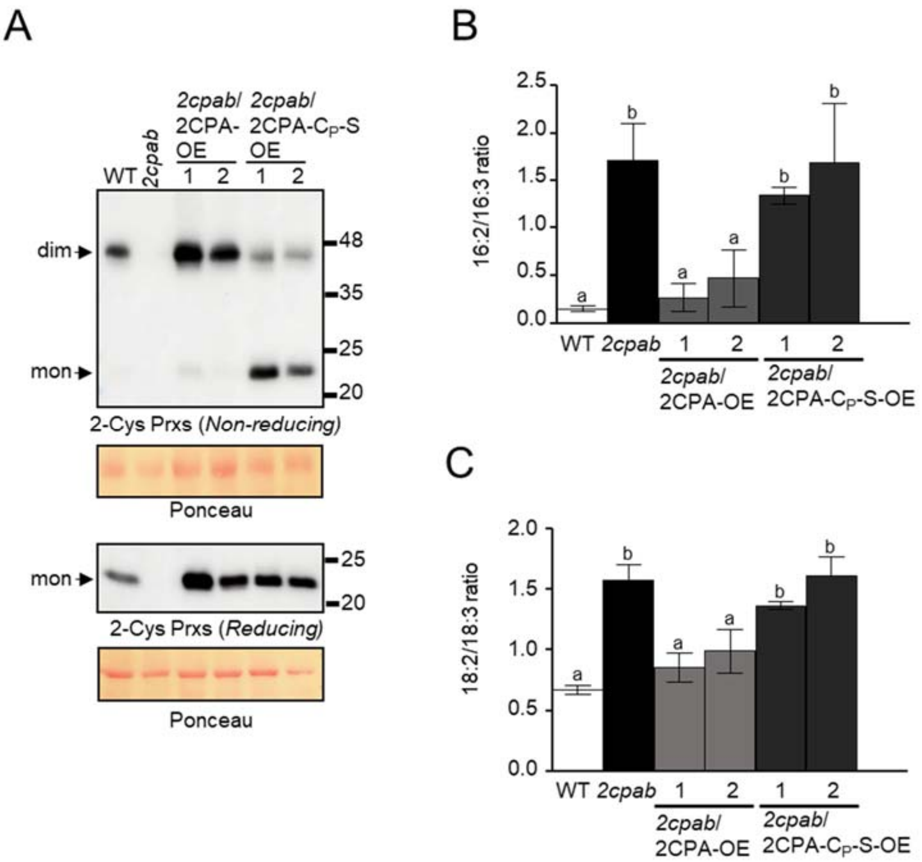

Supplemental Figure S2. Dienoic to trienoic fatty acid ratios in *2cpab*/2CPA-OE and *2cpab*/2CPA-CP-S transgenic lines. Wild-type (WT), the *2cpab* mutant and transgenic lines overexpressing 2-Cys Prx A (*2cpab*/2CPA-OE) or the mutant variant 2CPA-CP-S (*2cpab*/2CPA-CP-S) in the *2cpab* background were grown under long-day photoperiod for 4 weeks. **(A)** Western blot analysis of the levels of 2-Cys Prxs in WT, *2cpab*, and two (1 and 2) individuals of *2cpab*/2CPA-OE and *2cpab*/2CPA-CP-S transgenic lines. Protein extracts (15 µg), obtained from plants, were subjected to SDS-PAGE under non-reducing (top) or reducing (bottom) conditions, transferred to nitrocellulose filters, and probed with anti-2-Cys Prxs antibody. Even loading was monitored by Ponceau staining of the Rubisco large subunit. Monomeric (mon) and dimeric (dim) forms of 2-Cys Prxs are indicated by arrows. Molecular mass markers (kDa) are indicated on the right. 16:2/16:3 **(B)** and 18:2/18:3 **(C)** ratios in WT, *2cpab*, *2cpab/*2CPA-OE and *2cpab*/2CPA-CP-S rosette leaves grown as stated in **(A)**. Total leaf lipids were extracted, and fatty acid composition analysed as described in Materials and Methods. Data are means ± SD of four independent plants. Letters indicate significant differences by One-way ANOVA with a Tukey's Multiple Comparison Test ($p < 0.05$).

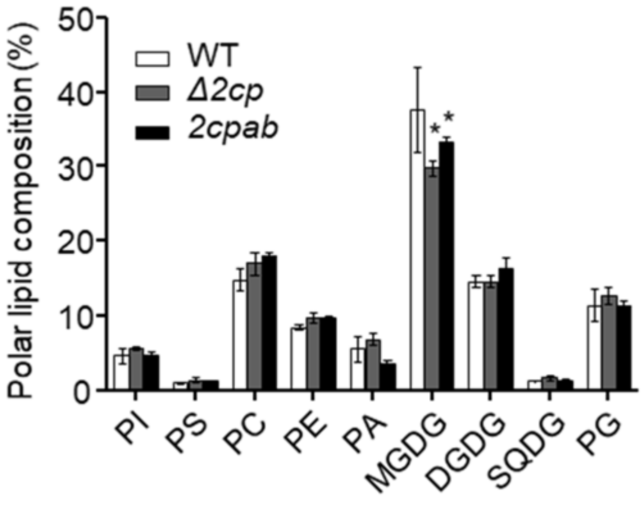

Supplemental Figure S3. Polar lipid composition of Arabidopsis rosette leaves from wild-type and 2-Cys Prxs deficient mutant plants. Total leaf lipids were extracted from rosette leaves of wild-type (WT) and the Δ*2cp* and *2cpab* mutant lines grown under long-day photoperiod for 4 weeks, and the different lipid classes were separated by TLC and quantified as described in Materials and Methods. Indicated lipids are digalactosyldiacylglcyerol (DGDG), monogalactosyldiacylglycerol (MGDG), phosphatidic acid (PA), phosphatidylcholine (PC), phosphatidylethanolamine (PE), phosphatidylglycerol (PG), phosphatidylinositol (PI), phosphatidylserine (PS), and sulfoquinovosyldiacylglycerol (SQDG). Data are means ± SD of three independent plants. Asterisks indicate significantly different ($p < 0.05$) to the WT according to two-way ANOVA with a Bonferroni post-test.

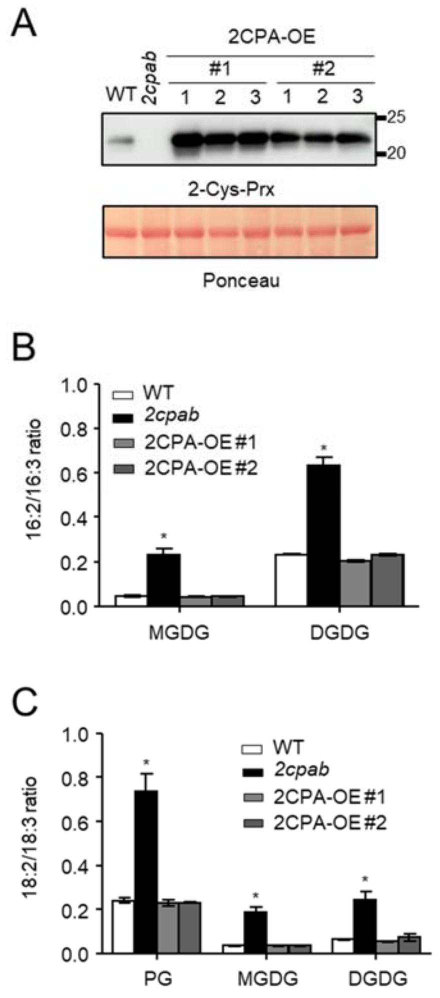

Supplemental Figure S4. Effect of the overexpression of 2-Cys Prx A on trienoic fatty acid levels in Arabidopsis leaves. Wild-type (WT), *2cpab* mutant, and transgenic lines overexpressing 2-Cys Prx A in the WT background (2CPA-OE) were grown under long-day photoperiod for 4 weeks. **(A)** Western blot analysis of the levels of 2-Cys Prxs in WT, *2cpab*, and three plants (1, 2 and 3) of two independent 2CPA-OE (#1 and #2) transgenic lines. Protein extracts (15 µg) were subjected to SDS-PAGE under reducing conditions, transferred to nitrocellulose filters, and probed with the anti-2-Cys Prxs antibody. Even loading was monitored by Ponceau staining of the Rubisco large subunit. Molecular mass markers (kDa) are indicated on the right. Dienoic to trienoic fatty acid ratios, 16:2/16:3 in galactolipids monogalactosyldiacylglycerol (MGDG) and digalactosyldiacylglycerol (DGDG) **(B)** and 18:2/18:3 in phosphatidylglycerol (PG), MGDG and DGDG **(C)**, from WT, *2cpab*, and 2CPA-OE rosette leaves grown as stated in **(A)**. Total leaf lipids were extracted, the different lipid classes were separated by TLC and fatty acid compositions analysed as described in Materials and Methods. Data are means ± SD of four independent plants. Asterisks indicate significantly different ($p < 0.05$) to WT according to two-way ANOVA with a Bonferroni post-test.

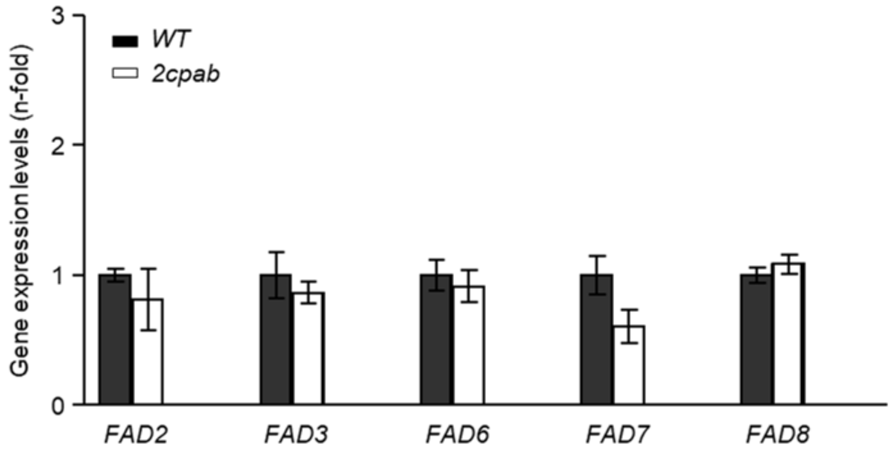

Supplemental Figure S5. RT-qPCR expression analysis of ω-6 and ω-3 desaturases in wild type and the *2cpab* mutant. Transcript levels of genes encoding ω-6 Fatty Acid Desaturase (FAD) 2 and 6 and ω-3 (FAD3, FAD7 and FAD8) desaturases in rosette leaves from wild-type (WT) and *2cpab* plants grown under long-day photoperiod for 4 weeks. For each gene, the levels of transcripts were normalized against two reference genes (Material and Methods) and referenced against the levels in WT plants (arbitrarily considered as 1). Values represent the mean ± SEM of three technical replicates. No significant differences were found between WT and *2cpab* (Student's t test; $p < 0.05$).

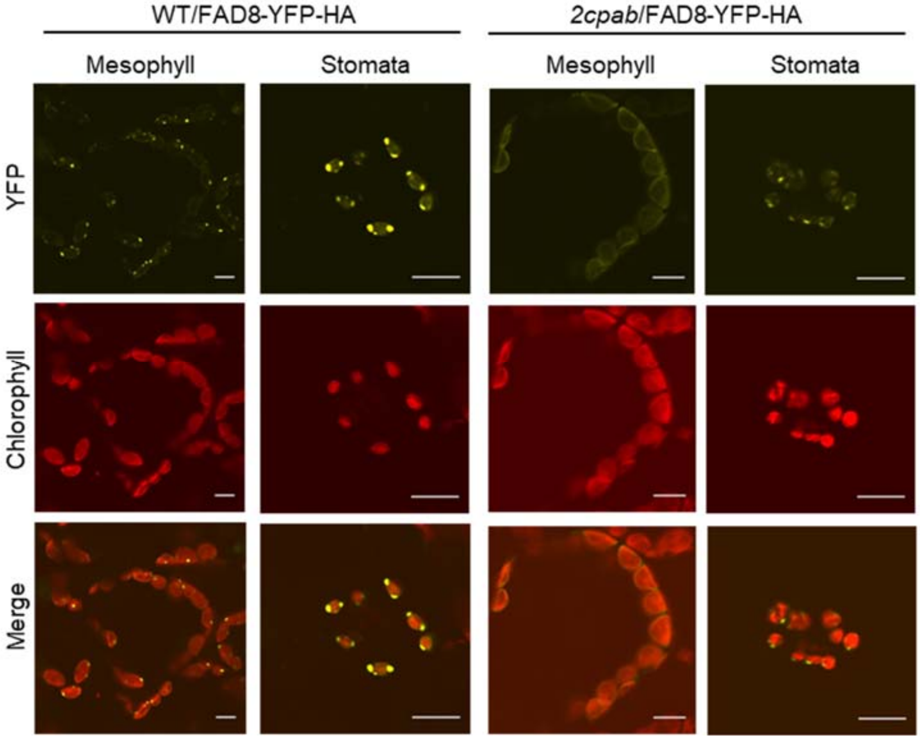

Supplemental Figure S6. Subcellular localization of FAD8 in rosette leaves from WT/FAD8-YFP-HA and *2cpab*/FAD8-YFP-HA plants. Transgenic lines overexpressing Fatty Acid Desaturase 8 (FAD8) fused to the YFP and HA tags in the wild-type (WT/FAD8-YFP-HA) and the *2cpab* (*2cpab*/FAD8-YFP-HA) genetic backgrounds were grown under long-day photoperiod for 4 weeks. Confocal images of mesophyll and guard cells (stomata) from rosette leaves of the indicated lines. Scale bars, 10 µm.

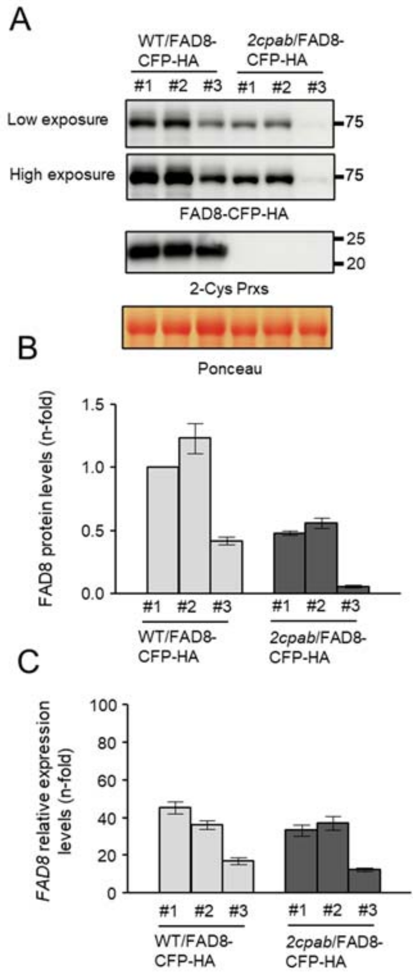

Supplemental Figure S7. Effect of the deficit of 2-Cys Prxs on the expression of FAD8 in rosette leaves from WT/FAD8-CFP-HA and *2cpab*/FAD8-CFP-HA transgenic plants. **(A)** Western blot analysis of the levels of Fatty Acid Desaturase 8 (FAD8) fused to the CFP and HA tags (FAD8-CFP-HA) and 2-Cys peroxiredoxins (Prxs) in protein extracts (15 µg) of rosette leaves from three independent transgenic lines (#1, #2 and #3) overexpressing FAD8-CFP-HA in the wild-type (WT/FAD8-CFP-HA) and the *2cpab* (*2cpab*/FAD8-CFP-HA) genetic backgrounds. Plants were grown under long-day photoperiod for 4 weeks. Even loading was monitored by Ponceau staining of the Rubisco large subunit (RbcL). Molecular mass markers (kDa) are indicated on the right. **(B)** Band intensities corresponding to FAD8-CFP-HA and RbcL were quantified (GelAnalyzer) and the contents of FAD8-CFP-HA, normalized to the levels of RbcL, are shown relative to the levels of the WT/FAD8-CFP-HA#1 (arbitrarily assigned a value of 1). Data are given as the mean ± SD. **(C)** Levels of transcript of *FAD8* determined by RT-qPCR in the same individuals as in **(A)**. Transcript levels were normalized against two reference genes (Materials and Methods) and referenced against the levels in WT plants (arbitrarily considered as 1). Values represent the mean ± SD of three replicates.

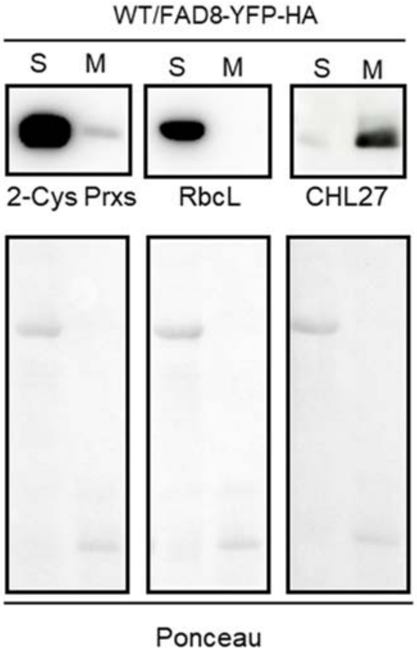

Supplemental Figure S8. Accumulation of 2-Cys Prxs in soluble and membrane protein fractions from Arabidopsis leaves. Transgenic plants overexpressing Fatty Acid Desaturase 8 (FAD8) fused to the YFP and HA tags (FAD8-YFP-HA) in the wild-type (WT) background (WT/FAD8-YFP-HA) were grown under long-day photoperiod from 4 weeks. Soluble (S) and membrane (M) protein fractions were obtained as described in Materials and Methods. Protein extracts (10 µg) were subjected to SDS-PAGE under reducing conditions, transferred to nitrocellulose filters, and probed with anti-2-Cys peroxiredoxins (Prxs) antibody, and anti-RbcL (large subunit of Rubisco) and anti-CHL27 (COPPER RESPONSE DEFECT 1) antibodies, as protein markers of the soluble (RbcL) and membrane fractions (CHL27). Even loading was monitored by Ponceau staining.

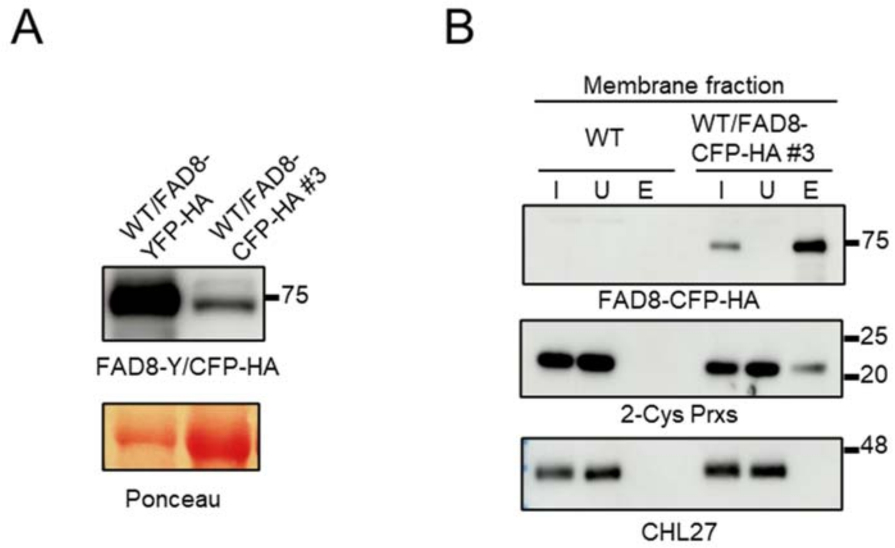

Supplemental Figure S9. Interaction of 2-Cys Prxs and FAD8-CFP-HA in Arabidopsis leaf membranes. Wild-type (WT) and transgenic plants overexpressing Fatty Acid Desaturase 8 (FAD8) fused to the YFP and HA tags (WT/FAD8-YFP-HA) or the CFP and HA tags (WT/FAD8-CFP-HA #3) were grown under long-day photoperiod for 4 weeks. **(A)** Western blot analysis of the levels of HA-tagged FAD8 in protein extracts (15 µg) of rosette leaves from WT/FAD8-YFP-HA and WT/FAD8-CFP-HA transgenic lines. Protein loading was monitored by Ponceau staining of the Rubisco large subunit. Molecular mass markers (kDa) are indicated on the right. **(B)** Membrane fractions obtained from formaldehyde-fixed leaves of WT and WT/FAD8-CFP-HA #3 plants, were subjected to immunoprecipitation using anti-HA antibody coupled to magnetics beads. Proteins preparations (2 µg) from the input (I) and unbounded (U) fractions and ~25% of the total eluted volume (E) were immunoblotted and probed with anti-HA, indicating the presence of FAD8-CFP-HA, anti-2-Cys peroxiredoxins (Prxs), and anti-CHL27 (COPPER RESPONSE DEFECT 1).

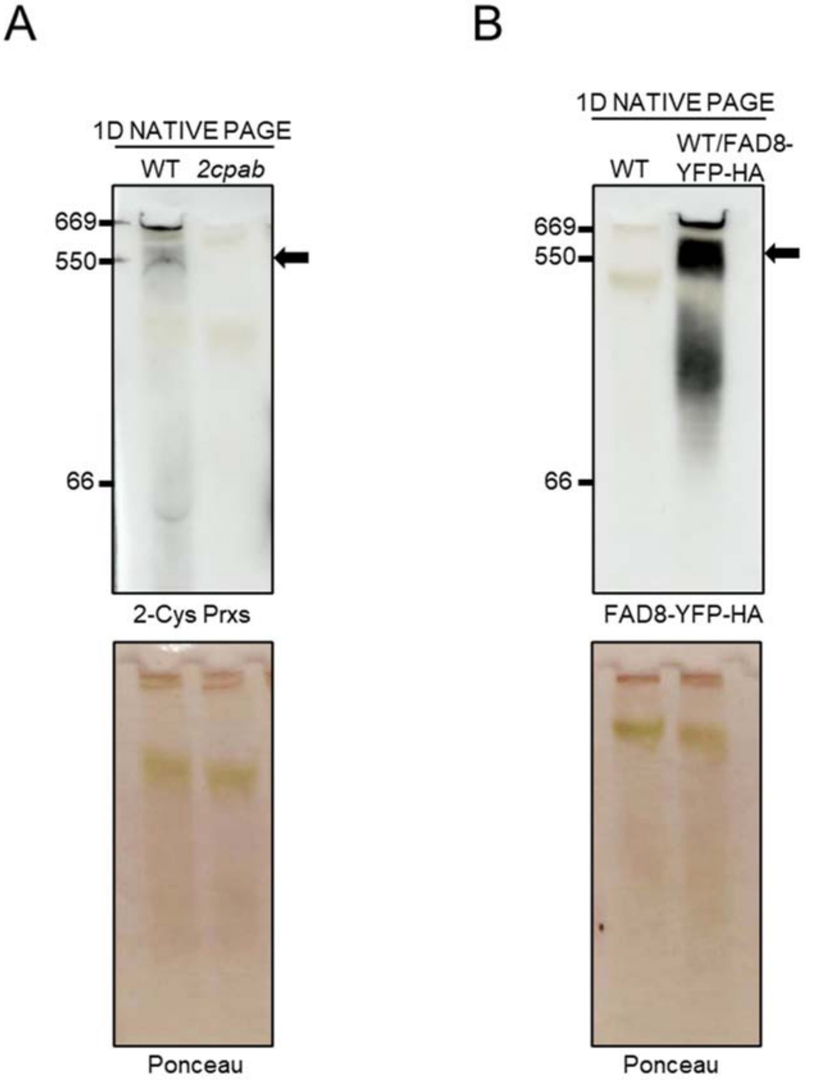

Supplemental Figure S10. Validation of the specificity of antibodies against 2-Cys Prxs and the HA-tag in Western blot analysis of native proteins. Wild-type (WT), *2cpab* and transgenic plants overexpressing Fatty Acid Desaturase 8 (FAD8) fused to the YFP and HA tags in the wild-type background (WT/FAD8-YFP-HA) were grown under long-day photoperiod for 4 weeks. Membrane fraction proteins (30 µg) from leaves of WT and *2cpab* plants **(A)** or WT and WT/FAD8-YFP-HA plants **(B)**, were separated in 1D-NATIVE-PAGE (5-12% acrylamide), transferred to nitrocellulose filters and probed with antibodies against 2-Cys peroxiredoxins (Prxs) **(A)** or the HA-tag **(B)**, which detects the FAD8-YFP-HA fusion protein. Molecular mass markers (kDa) are indicated on the left. Arrows indicate the band corresponding to 2-Cys Prxs **(A)** and FAD8-YFP-HA **(B)**. Even loading was monitored by Ponceau staining.
